# Safe immunosuppression-resistant pan-cancer immunotherapeutics by velcro-like density-dependent targeting of tumor-associated carbohydrate antigens

**DOI:** 10.1016/j.cell.2025.09.001

**Published:** 2025-09-25

**Authors:** Raymond W. Zhou, Paresh Kumar Purohit, Jai Hyun Kim, Sung-Uk Lee, Nicole Burshteyn, Delia Tifrea, Andres Cordon, Ani Grigorian, Barbara L. Newton, Robert A. Edwards, Michael Demetriou

**Affiliations:** 1 Department of Neurology, University of California, Irvine, Irvine, CA, USA; 2 GlyTR Therapeutics Inc., Irvine, CA, USA; 3 Department of Pathology & Laboratory Medicine, University of California, Irvine, Irvine, CA, USA; 4 Department of Microbiology and Molecular Genetics, University of California, Irvine, Irvine, CA, USA

**Keywords:** pan-cancer, immunotherapy, glycan-dependent T cell recruiter, tumor-associated carbohydrate antigens, β1,6GlcNAc-branched N-glycans, Tn antigen, siayl-Tn antigen, immunosuppression, tumor microenvironment, bispecific

## Abstract

Bispecific antibodies and chimeric antigen receptor T cells are some of the most potent cancer immunotherapeutics in clinical use, yet most cancers remain poorly targetable. High-affinity antibodies required to maximize killing detect low antigen expression in normal tissue, risking “on-target, off-cancer” toxicity. This compels identification of cancer-restricted cell-surface protein antigens, which are rare. Tumor-associated carbohydrate antigens (TACAs) are the most abundant and widespread cancer antigens known but are poorly targetable by antibodies. Here, we describe glycan-dependent T cell recruiter (GlyTR) pan-cancer immunotherapeutics that utilize high-avidity “velcro-like” lectin binding to kill cells with high but not low TACA expression. GlyTR1 and GlyTR2 bind immunosuppressive β1,6GlcNAc-branched N-glycans or multiple TACAs (Tn, sialyl-Tn, LacDiNAc, and GD2), respectively, overcome immunosuppressive mechanisms in the tumor microenvironment and trigger target-density-dependent T cell-mediated pan-cancer killing, yet they lack toxicity in mice with human-like TACA expression. Density-dependent lectin binding to TACAs provides highly potent and safe pan-cancer immunotherapeutics.

## Graphical abstract

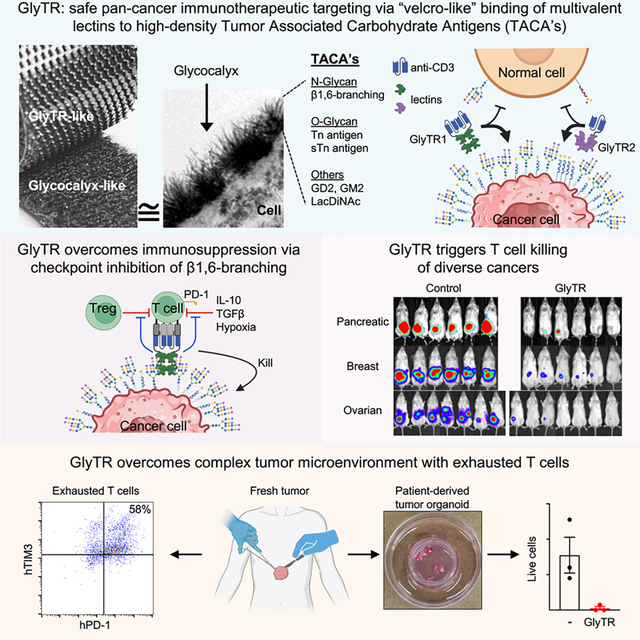

## Introduction

Bispecific antibodies and chimeric antigen receptor T cells (CAR T) potently reduce tumor burden in B cell-related malignancies. Both trigger T cell-mediated killing of cancer cells by targeting a cell-surface cancer antigen using modified antibodies. However, applying this therapeutic strategy to the majority of cancer types, particularly solid cancers, is limited by a lack of safe targetable protein antigens. High-affinity antibodies required for potent cancer killing also detect low target expression in normal tissue to generate potential on-target, off-cancer toxicity. This necessitates identification of antigens that are restricted to cancer and/or are expressed in non-essential cells like B cells, a major barrier to broader application. Moreover, even if safe antigens are identified, each antigen/cancer will require development of a different therapeutic, significantly increasing development time and costs. Thus, there remains a great need for single agents that have pan-cancer activity across numerous diverse liquid and solid cancers that lack on-target, off-cancer toxicity risk.

Many cell-surface cancer antigens are not proteins but rather complex carbohydrates and are termed “tumor-associated carbohydrate antigens” (TACAs).^1,2,3,4,5,6,7,8,9,10,11,12^ As virtually all cell-surface proteins are glycosylated, with each protein having multiple glycans, TACA density can be ~1,000–10,000-fold greater than typical protein antigens. Two well-described TACAs are β1,6GlcNAc-branched N-glycans and the Tn antigen, the latter an abnormally truncated O-glycan. β1,6GlcNAc-branched N-glycans constitute a small subset of the complex-type N-glycans expressed at the surface of normal human cells but are markedly upregulated by driver mutations in the receptor tyrosine kinase (RTK)/RAS/phosphoinositide-3-kinase (PI3K) signaling pathway.^3,4,5,6^ The Tn antigen is a single *N*-acetyl-galactosamine (GalNAc) α-*O*-linked to serine/threonine that is not typically expressed on the cell surface of normal human tissue but is expressed in ~90% of human carcinomas and many hematopoietic cancers secondary to cancer-associated defects in O-glycan biosynthesis.^7,8,9,10,11,12^ Aberrant over-expression of β1,6GlcNAc-branched N-glycans (β1,6-branching) is a critical driver of RTK signaling (up to ~100-fold),^13,14^ while both β1,6-branching and Tn antigen promote tumor growth, motility, invasion, and metastasis.^3,5,6,15,16,17,18,19^

As both markers and drivers of diverse cancers, β1,6-branching and Tn antigen provide excellent targets for antigen-specific immunotherapies. However, anti-glycan antibodies typically have affinities 1,000–100,000-fold lower than antibodies to peptide antigens. This is due to higher flexibility of glycans than peptides, absence of T cell help to B cells from lack of major histocompatibility complex (MHC) presentation of pure glycans, and attachment of glycans to a vast array of different proteins/lipids resulting in a non-uniform antigen.^20^ Indeed, anti-carbohydrate antibodies typically require additional peptide/lipid epitopes for high-affinity binding.^21^ Consistent with this, an antibody to β1,6-branching has never been generated, and only poor antibodies have been produced to pure Tn antigen.^22^ Although high-affinity antibodies to the glycopeptide Tn-MUC1 have been used to generate effective CAR T cells, the antibody was not reactive with ~50% of tested Tn^+^ cancers.^23^ Moreover, targeting glycopeptides rather than pure glycan antigens also increases the risk of tumor escape, a consequence of potential protein mutation on top of alterations to the glycan.

The inability to generate an antibody to β1,6-branching and effective antibodies to pure Tn antigen has prevented effective targeting of these well-established tumor-associated antigens. To address this issue, we envisioned a class of immunotherapeutics that utilize sugar-binding proteins (lectins) that have well-established specificity, rather than antibodies, to target glycan antigens. We have termed this “glycan-dependent T cell recruiter” (GlyTR, pronounced “glitter”). GlyTR bispecific proteins fuse a carbohydrate-recognition domain (CRD) from a lectin to a single-chain variable fragment (scFv) from an antibody targeting CD3. Lectins utilize high binding avidity (velcro-like binding) to achieve specificity for glycan targets. This is in distinction to antibodies, where high affinity (key-lock binding) achieves specificity. High-avidity binding of lectins results from the high density of glycan targets on the cell surface and multiple CRDs in the lectin. Here, we test the hypothesis that multivalent GlyTR immunotherapeutics should allow safe pan-cancer activity by targeting high-TACA density cancer cells while evading the risk of on-target, off-cancer toxicity by ignoring lower-expressing normal tissue.

## Results

### GlyTR1 uses L-PHA for high-avidity targeting of β1,6GlcNAc-branched N-glycans

To target β1,6-branching, we chose L-PHA (*Phaseolus vulgaris, leukoagglutinin*), a tetrameric plant lectin that requires β1,6-branching for binding, as targeted deletion of *Mgat5* or earlier Golgi enzymes (e.g., *Mgat1* and *Mgat2*) is also required for biosynthesis block binding.^24,25,26^ Flow cytometry with L-PHA confirmed high target density in a wide diversity of solid and liquid cancers, with binding up to ~25-fold higher than normal T cells (Figure S1A). Moreover, L-PHA binding was similarly elevated (~6–12 times) in all randomly selected patient-derived tumors and cancer stem cells (CSCs) (Figures S1B and S1C; Table S1).

**Figure 1.**
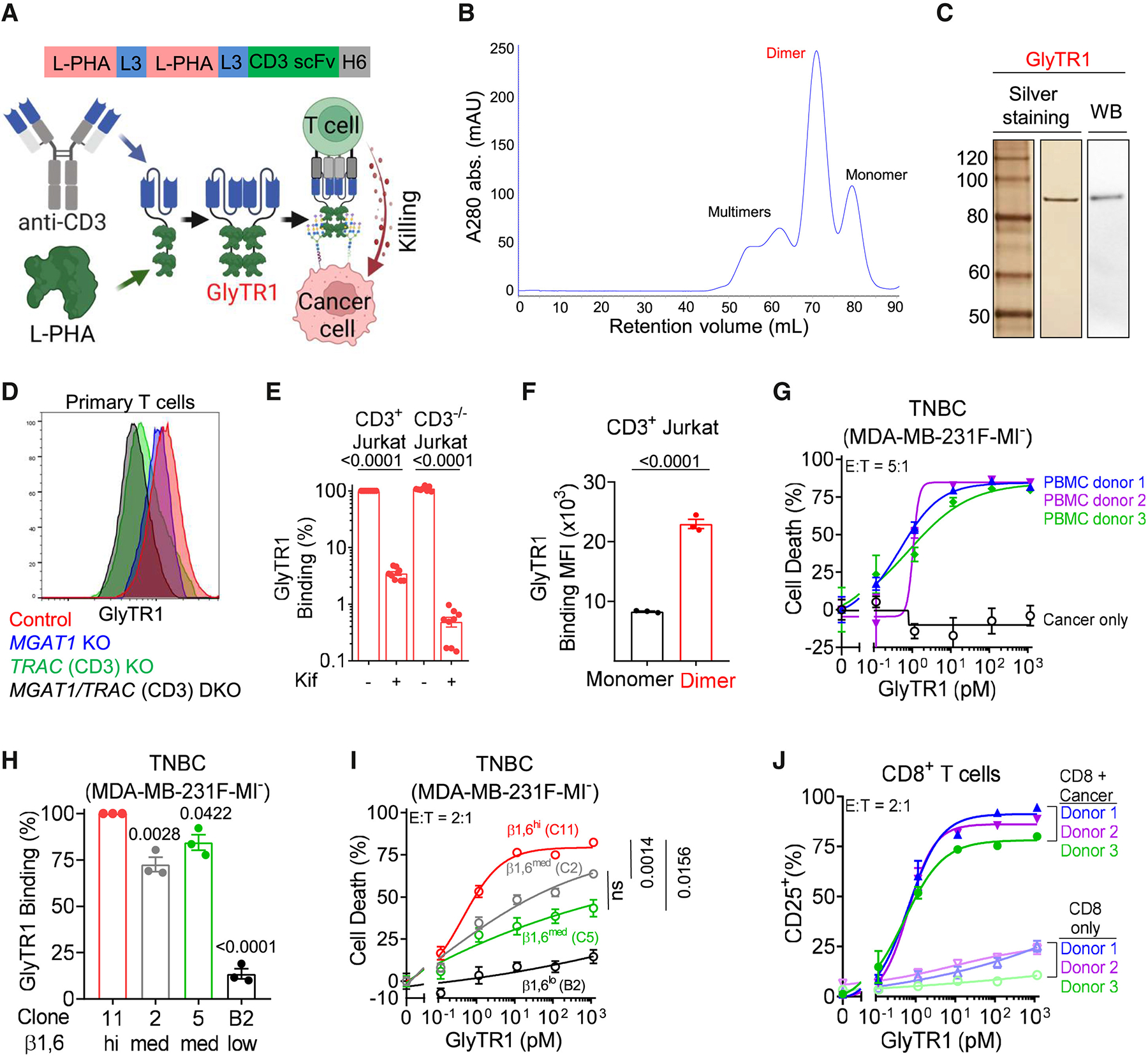
Density-dependent targeting of β1,6GlcNAc-branched N-glycans by optimized GlyTR1 bispecific protein (A) Optimized dimeric GlyTR1 bispecific protein design. L3: 3x flexible linker; scFv: single-chain variable fragment; H6: 6x histidine tag. (B) Size exclusion chromatography of GlyTR1 after affinity purification. (C) Silver staining and immunoblots (*n* ≥ 3) of GlyTR1 after reducing SDS-PAGE. (D) Flow cytometry of GlyTR1 binding to the indicated human primary T cells (see also Figure S1L). *MGAT1*^−/−^ was identified as ConA^high^. (E and F) Flow cytometry of GlyTR1 binding to the indicated Jurkat T cells treated with/without kifunensine (kif). (G) Flow cytometry of cancer cell death by GlyTR1 following 3-day co-culture of TNBC cells with/without PBMC from *n* = 3 donors and effector-to-target (E:T) ratio at 5:1. (H and I) Flow cytometric assessment of GlyTR1 binding and primary human CD8^+^ T cell triggered killing (E:T = 2:1; pooled from 3 independent experiments) of MHC class I-deficient (B2M^−/−^) MDA-MB-231F-MI^−^ breast cancer cell clones. Clone B2 is *MGAT5*^−/−^. (J) Flow cytometry of GlyTR1 triggered primary human CD8^+^ T cell activation with or without clone 11 MDA-MB-231F-MI^−^ TNBC cells with an E:T ratio at 2:1. Data are mean ± SEM from *n* ≥ 3. *p* values by repeated measure one-way ANOVA with Sidak’s multiple comparisons test (E and H), two-tailed paired Student’s t test (F), or nonlinear regression (I). See also Figure S1 and Table S1.

To first develop a GlyTR1 protein targeting β1,6-branching, we linked a single L-PHA domain to an scFv domain specific to the human CD3 protein (OKT3 clone) (Figure S1D). After expression in ExpiCHO-S cells, size-exclusion chromatography (SEC) revealed that GlyTR1^L-PHAxCD3^ was predominantly a dimer of ~100 kDa versus 55 kDa predicted (Figure S1E) and thus contained two L-PHA and two anti-CD3 domains. Dimeric GlyTR1^L-PHAxCD3^ specifically bound to both human CD3 and β1,6-branching, as blocking the latter with the mannosidase I inhibitor kifunensine (kif)^27^ eliminated binding in non-CD3-expressing K562 cells but only reduced binding in CD3-expressing Jurkat T cells (Figure S1F). To confirm that two L-PHA domains serve to enhance target binding and cancer killing, we deleted the first five amino acids of the L-PHA domain in dimeric GlyTR1^L-PHAxCD3^, which are required for initiating multimerization but are distant from the carbohydrate-binding site.^28^ This significantly reduced binding to β1,6-branching and the ability to kill RPMI8226 multiple myeloma cells relative to dimeric GlyTR1^L-PHAxCD3^ (Figures S1G and S1H). Given this, we further increased the binding avidity of GlyTR1 by generating GlyTR1^L-PHA(2)xCD3^ with two L-PHA domains linked in tandem (Figure 1A), hereafter simplified as GlyTR1. Blotting and SEC revealed that GlyTR1 is ~50%–70% dimer, with the rest monomer (~20%–30%) or larger multimers (~20%–30%) (Figures 1B, 1C, and S1I). Dimer formation was stable, as re-running the dimer fraction on SEC revealed that >99% remained as a dimer (Figure S1J). Specificity of dimeric GlyTR1 to both human CD3 and β1,6-branching was confirmed with binding to *MGAT1*-deficient HEK293S cells (Figure S1K) and primary human T cells with/without *MGAT1* knockout (β1,6-branching deficient) and/or *TRAC* knockout (T cell receptor [TCR], CD3-deficient) (Figures 1D and S1L). *MGAT1* knockout blocked GlyTR1 binding to HEK293S cells (Figure S1K) while only minimally reducing GlyTR1 binding to primary T cells with, but not without, CD3 (Figures 1D and S1L). This indicates that GlyTR1 binding to primary T cells is dominated by the anti-CD3 domain, while the L-PHA domain has little impact. The opposite was observed for GlyTR1 binding to Jurkat leukemia T cells, where blocking β1,6-branching with kif markedly reduced binding while CD3 deficiency had little impact on binding (Figure 1E). This difference is consistent with the ~3× higher β1,6-branching in Jurkat over primary T cells (Figure S1A) driving density-dependent β1,6 binding of GlyTR1. Directly comparing the monomeric (two L-PHA domains) and dimeric (four L-PHA domains) fractions of GlyTR1 revealed significantly higher binding of the latter to Jurkat T cells (Figure 1F), further confirming that increasing the number of TACA-binding domains within GlyTR1 proteins leads to higher-binding avidity. Similarly, dimeric GlyTR1 (four L-PHA domains) also bound to Jurkat T cells significantly better than dimeric GlyTR1^L-PHAxCD3^ (two L-PHA domains) (Figure S1M), leading to a >3,000-fold increase in cancer cell killing activity (Figure S1N). Dimeric GlyTR1 killing of MDA-MB-231F triple-negative breast cancer (TNBC) cells was similar across three different peripheral blood mononuclear cell (PBMC) donors (Figures 1G and S1O), with 50% of maximal killing at an effector (CD8^+^ T cell) to tumor cell ratio of ~1:4 (Figure S1P). Killing was human T cell dependent, as there was little killing in the absence of human T cells (Figures 1G and S1O) or in the presence of mouse T cells (Figure S1Q).

To confirm that small changes in GlyTR1-binding avidity lead to synergistic changes in killing activity, we isolated/generated clones of MDA-MB-231F TNBC cells lacking β2-microglobulin that had high, intermediate, and low levels of β1,6-branching (Figures 1H and S1R), the latter by CRISPR-Cas9 knockout of *MGAT5*. Residual low-level binding in *MGAT5* knockout cells likely arises from *MGAT5b*, a β1,6-N-acetylglucosaminyltransferase typically expressed in the brain. Major Histocompatibility Complex class I (MHC class I) antigen presentation is not required for killing, as CD8^+^ T cells readily killed MDA-MB-231F-MI^−^ TNBC cells deleted for β2-microglobulin (Figure 1I). Reductions in GlyTR1 binding of ~15%–30% resulted in marked reductions in killing activity, while low target expression (*MGAT5* knockout) resulted in little appreciable killing (Figures 1H and 1I). Thus, small changes in binding avidity, either through altering the number of CRDs or target glycan density, both significantly impact GlyTR1-triggered T cell killing of target cancer cells.

L-PHA, like other lectins, is cytotoxic to cells at ~5 μg/mL concentrations^23^; however, this is >5,000-fold higher than the ~0.5–1 ng/mL of GlyTR1 required to robustly trigger T cell-dependent killing. At 2–10 μg/mL, L-PHA is also a T cell mitogen, but this is also >1,000-fold higher than GlyTR1 concentrations that trigger T cell activation in the presence but not absence of cancer (Figures 1J and S1S–S1U). Consistent with this, reducing β1,6-branching by ~75% in T cells via kif pre-treatment^27^ did not reduce GlyTR1 cancer killing (Figures S1V and S1W). Coupled with minimal binding of the L-PHA domain in GlyTR1 to primary T cells (Figures 1D and S1L), these data indicate that L-PHA binding to T cells plays little direct role in high-potency T cell killing triggered by GlyTR1 and is consistent with targeting high-TACA-density cancer cells but not low-density T cells for killing.

### GlyTR2 uses CD301 for high-avidity targeting of the Tn/ sTn antigen and related TACAs

To generalize the concept of using lectins for high-avidity targeting of TACAs, we generated the GlyTR2 bispecific protein by utilizing the human CD301 lectin (CLEC10A, macrophage galactose lectin) that binds five different TACAs.^29,30,31,32,33,34,35^ CD301 is a transmembrane lectin expressed in macrophages and dendritic cells (DCs) that functions as a pattern recognition receptor for non-self antigens, particularly the invertebrate glycan LacDiNAc (GalNAcβ1,4GlcNAc),^29,30^ as well as multiple TACAs. CD301 binds to GalNAc with exposed 3- and 4-hydroxyl groups, structures typified by the Tn cancer antigen as well as three other well-known TACAs, namely sialyl-Tn (sTn)^31,32^ and the gangliosides GD2 and GM2^33^ but not common glycans.^32,33,34,35^ Although mammalian cells generally do not express LacdiNAc, expression is often induced in many human cancers, providing a fifth TACA targetable by CD301.^36^ Red blood cells (RBCs) express two glycans with terminal GalNAc, namely the blood group A antigen and globoside (globotetraosylceramide-4 [Gb4] or P antigen), the latter common to all RBC except for rare mutations.^37^ However, CD301 is expressed normally in humans without inducing toxicity and failed to bind blood group A positive RBC or blood vessels at concentrations that readily bound breast cancer.^29^ Indeed, analysis of high-density glycan microarrays revealed that CD301 binds Tn antigen with ~10-fold higher affinity than blood group A-glycan.^38^

**Figure 2.**
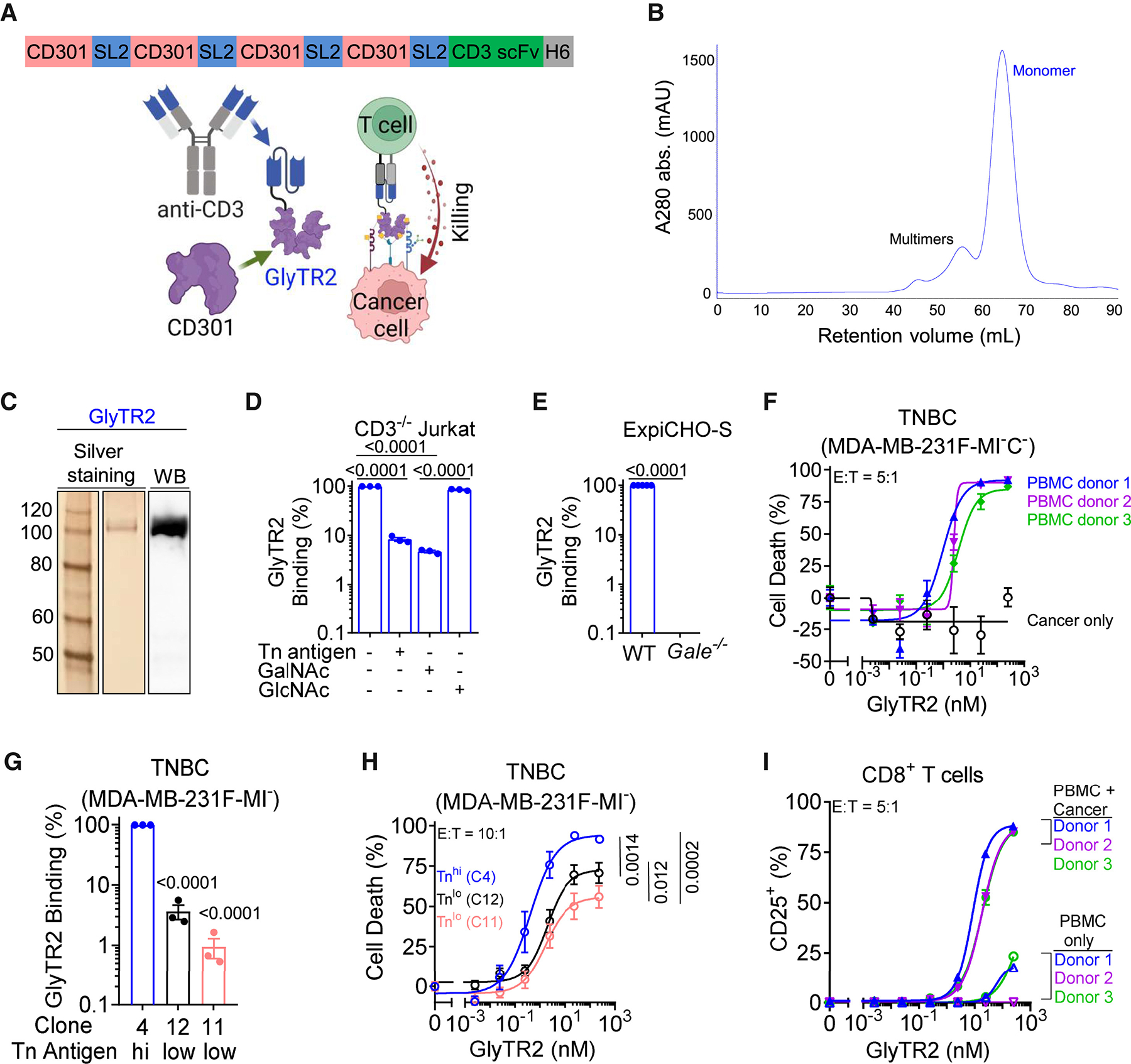
Density-dependent targeting of five N-acetylgalactoasmine-containing TACAs by optimized GlyTR2 bispecific protein (A) Optimized monomeric GlyTR2 bispecific protein design targeting Tn/sTn antigen, GD2, GM2, and LacDiNAc. SL2: 2× stiff linker; scFv: single-chain variable fragment; H6: 6× histidine. (B) Size exclusion chromatography analysis of GlyTR2 after affinity purification. (C) Silver staining and immunoblots (*n* ≥ 3) of GlyTR2 after reducing SDS-PAGE. (D and E) Flow cytometry of GlyTR2 binding to the indicated cells with/without soluble Tn antigen (0.25 mM), GalNAc (1 mM), GlcNAc (1 mM), or *Gale* knockout. Jurkat T cells are naturally COSMC^−/−^. (F) Flow cytometry of cancer cell death by GlyTR2 following 3-day co-culture of COSMC^−/−^ (clone 4) TNBC cells with/without PBMC from *n* = 3 donors at E:T = 5:1. (G and H) Flow cytometric assessment of GlyTR2 binding and primary human CD8^+^ T cell triggered killing (E:T = 10:1; pooled from 3 independent experiments) of MHC class I-deficient (B2M^−/−^) MDA-MB-231F-MI^−^ breast cancer cell clones. Clone 4 is COSMC^−/−^. (I) Flow cytometry of GlyTR2 triggered CD8^+^ T cell activation in PBMCs cultured with or without COSMC^−/−^ TNBC (clone 4) cells with E:T = 5:1. Data are mean ± SEM from *n* ≥ 3. *p* values by repeated measure one-way ANOVA with Sidak’s multiple comparisons test (D and G), two-tailed paired Student’s t test (E), or non-linear regression (H). See also Figure S2.

For the GlyTR2 bispecific protein, we linked the extracellular domain of human CD301 with the same anti-CD3 scFv (OKT3) domain used in GlyTR1. However, this protein was unable to be expressed in ExpiCHO-S cells, presumably because of protein misfolding. The CD301 extracellular domain consists of a neck region and a single CRD,^32,39^ with the former promoting trimerization.^32,40^ Therefore, we fused a single CD301 CRD without most of the neck region to the OKT3 scFv to generate GlyTR2^CD301xCD3^ (Figure S2A). This was readily expressed in ExpiCHO-S cells and bound Tn^high^ Jurkat-TCRβ^−/−^ leukemic T cells (Figure S2B), which lack CD3 but express maximal levels of Tn antigen due to a natural mutation of the chaperone protein COSMC required to extend O-linked GalNAc with galactose.^7^ Point mutation of 5 amino acids critical for sugar and calcium-binding in CD301, namely Gln267Gly, Asp269Gly, Glu280Gly, Asn292Gly, and Asp293Gly (NCBI RefSeq: NP_878910.1),^41^ abolished binding of mutGlyTR2^CD301xCD3^ to Tn^high^ Jurkat-TCRβ^−/−^ leukemic T cells (Figure S2B). Consistent with multiple TACA-binding domains enhancing binding avidity, GlyTR2^CD301(3)xCD3^ with three CD301 domains was superior to GlyTR2^CD301xCD3^ at binding to Tn^high^ Jurkat-TCRβ^−/−^ leukemic T cells (Figure S2B). GlyTR2^CD301(3)xCD3^ but not soluble human CD301 bound to CD4^+^ T cells, while neither protein bound significantly to CD19^+^ B cells (Figure S2C), confirming GlyTR^CD301(3)xCD3^ binds CD3 in T cells. By contrast, GlyTR2^CD301(3)xCD3^ and CD301 both bound to Tn^high^ Jurkat-TCRβ^−/−^ leukemic T cells (Figure S2C). Soluble Tn antigen (GalNAcα-Ser) and/or GalNAc, but not related sugars galactose and GlcNAc, blocked binding of GlyTR2^CD301(3)xCD3^ to Tn^high^ Jurkat-TCRβ^−/−^ cells, confirming binding specificity (Figure S2D). Although CD301 has been reported to bind CD45 (RA, RB, and RC) on normal T and B cells,^42^ we find that CD301 does not significantly bind to resting B cells or resting/activated T cells at concentrations that readily bind to Tn^+^ cancer cells (Figures S2C and S2E). However, the SEC indicated that GlyTR2^CD301(3)xCD3^ was predominantly made up of multiple multimers that were poorly resolved (Figure S2F), which may negatively impact activity, safety, and manufacturing consistency. Therefore, to reduce the potential for multimerization while maintaining a high number of TACA-binding domains, we added a fourth CD301 domain but replaced the flexible linkers (GGGGS(3)) separating individual CD301 domains with stiff linkers (AEAAAKA(2)) (GlyTR2^slCD301(4)xCD3^, Figures 2A and S2F). Indeed, SEC revealed that GlyTR2^slCD301(4)xCD3^, hereafter simplified to GlyTR2, was ~90% monomer and was stable, as re-running the monomer fraction on SEC revealed a single peak (Figures 2B, 2C, and S2F). Although monomeric GlyTR2 (stiff linkers, four CD301 domains) bound to Tn^high^ Jurkat-TCRβ^−/−^ leukemic T cells similar to multimeric GlyTR2^CD301(3)xCD3^ (flexible linkers, three CD301 domains), it bound significantly better to a wide diversity of lower target-expressing tumor cell lines (Figures S2G and S2H). As with GlyTR2^CD301(3)xCD3^, soluble Tn antigen and GalNAc but not GlcNAc readily blocked binding of GlyTR2 to Tn^high^ Jurkat-TCRβ^−/−^ leukemic T cells (Figure 2D). Similarly, GlyTR2 binding to ExpiCHO-S cells was eliminated by preventing UDP-GalNAc production required for O-glycan synthesis via genetic deletion of UDP-glucose 4-epimerase (*Gale*) (Figure 2E). Consistent with CD301-binding data,^29^ GlyTR2 did not significantly bind RBC, including A-positive RBC, at concentrations that robustly bound to Tn^high^ Jurkat-TCRβ^−/−^ leukemic T cells (Figure S2I).

To assess target density-dependent killing by GlyTR2, we evaluated three clones of MHC class I-deficient MDA-MB-231F-MI^−^ TNBC cells with modest or maximal Tn expression, and the latter was generated by deletion of COSMC^7^ (i.e., MDA-MB-231F-MI^−^C^−^, Figure S2J). T cell-induced killing of Tn^hi^ TNBC cells by GlyTR2 was similar across three different PBMC donors (Figure 2F). GlyTR2 triggered killing of TNBC cells in proportion to target density (Figures 2G and 2H), required T cells for killing (Figure 2F), and induced robust T cell activation in the presence but not absence of target-positive cancer cells (Figures 2I and S2K). Given these data, GlyTR2 (four CD301 domains, stiff linkers) was selected for further characterization.

### High-potency pan-cancer killing by optimized GlyTR1 and GlyTR2

**Figure 3.**
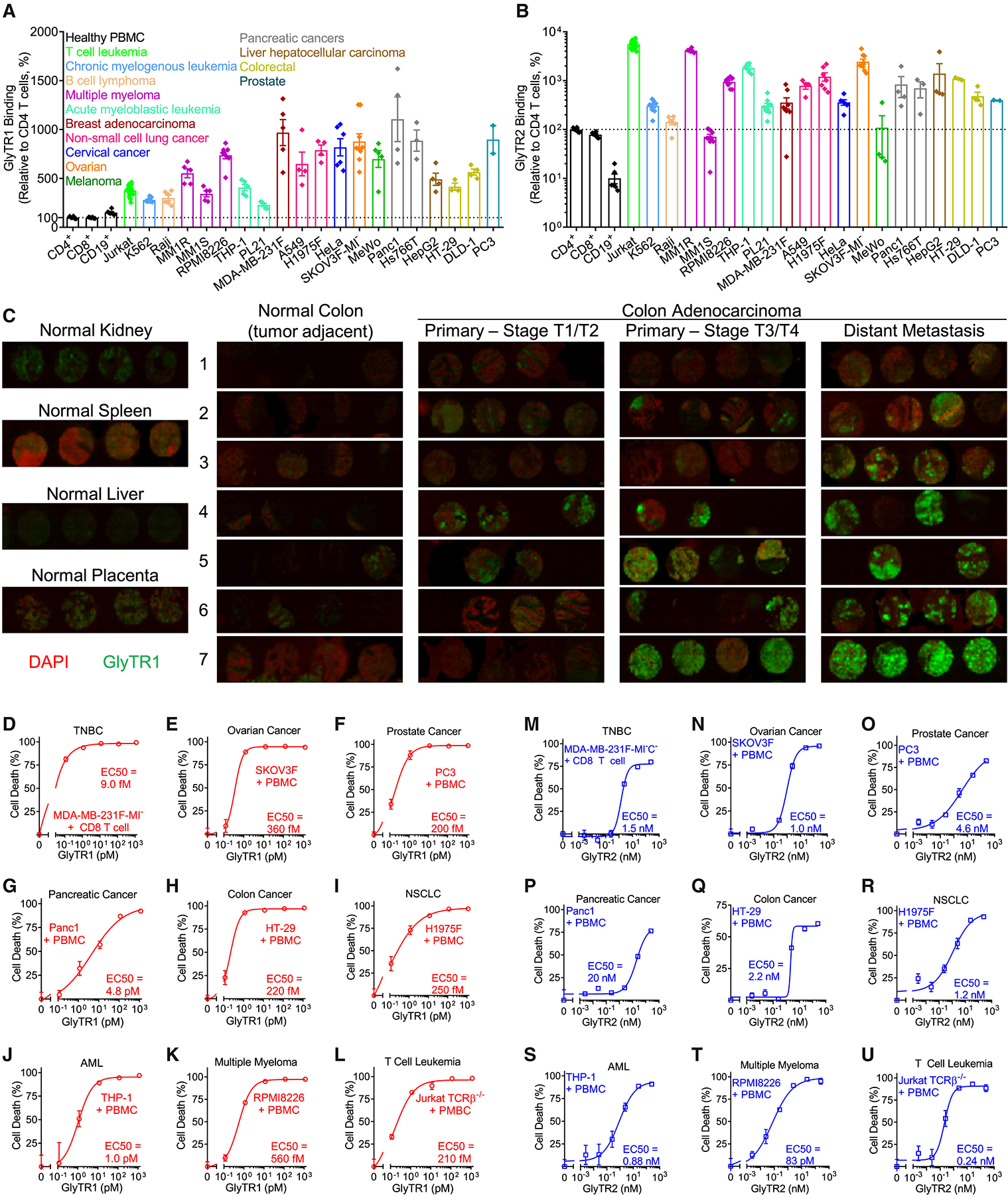
Target expression and pan-cancer killing by GlyTR1 and GlyTR2 (A and B) Flow cytometry of GlyTR1 (A) and GlyTR2 (B) binding to primary lymphocytes and the indicated cancer cell lines. Data are pooled from multiple independent experiments (*n* ≥ 3, except *n* = 2 for PC3 cells) with mean ± SEM normalized to healthy human CD4^+^ T cells at 100% (horizontal dotted line). (C) GlyTR1-FITC staining of a human colorectal carcinoma progression TMA. Each picture has 4 cores from one individual. Raw image in Data S1. (D–U) Flow cytometry of GlyTR1 (D–L) or GlyTR2 (M–U) triggered cancer cell death via co-culturing the indicated cancer cells with primary human CD8^+^ T cells (D and M) or PBMC (E–L and N–U) for 3 days and with E:T ratios at 10:1 (E, L–N, and U) or 20:1 (D, F–K, and O–T). EC_50_: half maximal effective concentration. Data are the mean ± SEM of 3 biological replicates. See also Figure S3 and Data S1.

Consistent with the known broad expression of β1,6-branching and Tn antigen in cancer, flow cytometry confirmed that both dimeric GlyTR1 and monomeric GlyTR2 displayed high levels of binding to a wide diversity of solid and liquid cancers relative to normal lymphocytes (Figures 3A and 3B). Most cancer lines co-expressed both targets with high density; however, the absolute number of binding sites per cell could not be determined, as saturation binding could not be achieved even at 1.25 mg/mL L-PHA, ~1,000,000-fold higher than required for GlyTR1 killing. GlyTR1-fluorescein isothiocyanate (FITC) immunofluorescence of a colon adenocarcinoma progression tissue microarray (TMA) demonstrated little binding to multiple normal tissues (kidney, spleen, liver, placenta, and colon), but increasing binding with disease stage and progression to metastatic disease (Figure 3C; Data S1). This is consistent with β1,6-branching being highest in metastatic disease.^3,5,6,15,16,17^

Both GlyTR1 and GlyTR2 bispecific proteins potently induced T cell-dependent killing of all tested cancer types with high target density *in vitro*, including TNBC, ovarian, prostate, pancreatic, colon, non-small cell lung cancer (NSCLC), acute myelogenous leukemia (AML), multiple myeloma, and T cell leukemia (Figures 3D–3U). At concentrations that readily induced cancer cell killing, GlyTR1 and GlyTR2 did not kill normal primary cultured cells, including T cells (Figures S3A–S3D), human renal epithelial cells, hepatocytes, prostate epithelial cells, and colon epithelial cells (Figures S3E–S3K). Consistent with this, GlyTR1 and GlyTR2 binding to these primary human cell lines was significantly lower than the SKOV3 ovarian cancer cell line (Figures S3L and S3M). As primary cells (other than T cells) required proprietary media with growth factors that can activate ERK/Map kinase, a pathway that upregulates β1,6-branching,^3,4,5,6^ these data likely overestimate GlyTR1 binding and killing risk to healthy cells *in vivo*.

**Figure 4.**
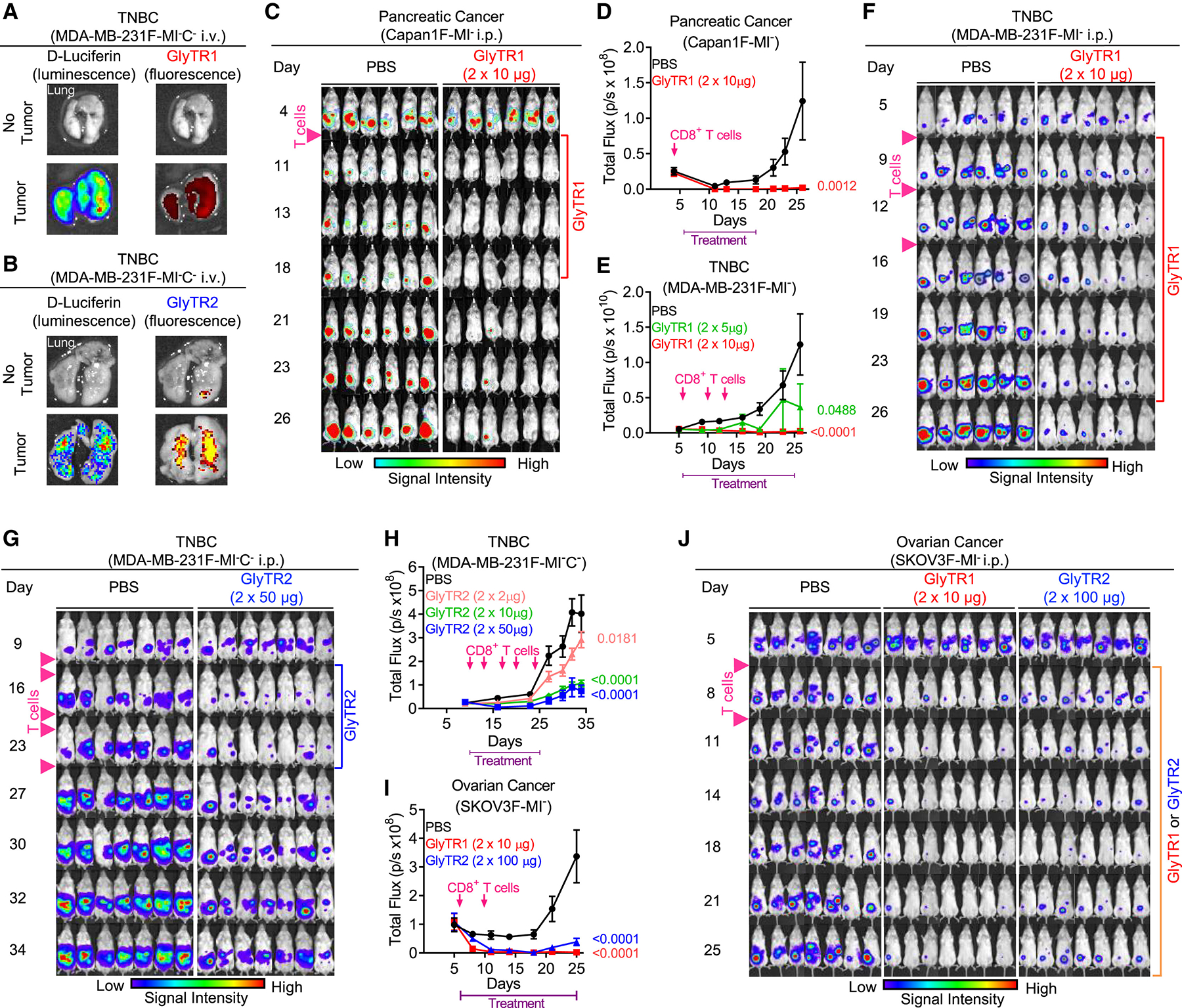
Tumor regression by GlyTR1 and GlyTR2 in xenogeneic-humanized mouse models (A and B) NSG mice with/without breast cancer metastasis from tail vein injection were injected i.v. with fluorophore (VivoTag680XL)-labeled GlyTR1 (A) or GlyTR2 (B), and extracted lungs were imaged for luminescence (tumor) and fluorescence (GlyTR1 or GlyTR2). (C–J) NSG mice were injected intraperitoneally (i.p.) with the indicated cancer cells on day 0 (*n* = 6 or 7 per group), then starting on day 4 (C and D), 6 (E, F, I, and J) or 10 (G and H) were injected i.p. with CD8^+^ T cells every 3–4 days (as indicated) (except the 2nd–5th injections in (G) and (H) with 2 × 10^6^ CD8^+^ T cells) as well as with/without i.p. GlyTR1 or GlyTR2 twice daily. Data points represent mean ± SEM. *p* values by linear regression analysis (D, E, H, and I). See also Figure S4.

Syngeneic mouse models could not be used to assess *in vivo* activity, as the two GlyTR proteins do not bind mouse CD3. Rather, we utilized humanized NSG mice and xenogeneic transplants of luciferase-expressing and MHC class I-deficient pancreatic ductal adenocarcinoma (PDAC) (Capan1F-MI^−^), TNBC (MDA-MD-231F-MI^−^ or MDA-MB-231F-MI^−^C^−^), and ovarian cancer (SKOV3F-MI^−^) cell lines. β1,6-branching was similarly high in the three lines, while Tn antigen expression was high in COSMC-deficient MDA-MB-231F-MI^−^C^−^ and intermediate in SKOV3F-MI^−^ cells (Figure S4A). MHC class I deficiency limits allogeneic killing by non-matched CD8^+^ T cells and was necessary for robust tumor growth in NSG mice humanized with CD8^+^ T cells. Both GlyTR proteins preferentially accumulated in lungs with, but not without, metastatic TNBC (Figures 4A and 4B) and dose-dependently induced robust tumor regression in the PDAC, TNBC, and/or ovarian intraperitoneal (i.p.) cancer models in CD8^+^ T cell-humanized NSG mice (Figures 4C–4J). The ovarian model was independently replicated at the National Cancer Institute (Figures S4B and S4C). As NSG mice with human T cells develop graft-versus-host disease (GvHD) starting at ~3–4 weeks, leading to mortality, long-term survival analysis was not assessed. In a metastatic model using intravenously (i.v.)-injected MDA-MB-231F-M1^−^C^−^ TNBC cells, GlyTR1 also showed similar activity (Figure S4D). To assess activity against liquid cancers, we utilized TCRβ^−^ Jurkat leukemia T cells, which lack CD3. 2 weeks after i.v. tumor inoculation, NSG mice were humanized with PBMC, treated with vehicle, GlyTR1, or GlyTR2^CD301(3)xCD3^ daily for 7 days, and then sacrificed. Both reduced tumor burden in the spleen up to 90% after 1 week of treatment (Figures S4E and S4F). Consistent with T cell-induced killing of cancer cells, the number of normal human splenic CD8^+^ > CD4^+^ T cells was significantly increased in the GlyTR-treated mice (Figures S4G and S4H).

To explore GlyTR activity in a syngeneic model with a normal immune system, a mouse-specific GlyTR1 (mGlyTR1) was generated using the 2C11 anti-mouse CD3 scFv; however, this was >10,000-fold less potent than hGlyTR1 (EC_50_ ~10 nM versus ~500 fM, Figure S4I). Moreover, mGlyTR1 robustly activated mouse T cells without cancer at these elevated concentrations (Figure S4J). This disparity may have arisen from (1) differences in the anti-CD3 domains or (2) mouse T cells being less responsive than human T cells to GlyTR1. To address these two possibilities, we replaced mouse CD3ε,δ,γ with human CD3ε,δ,γ (Figures S4K and S4L). However, hGlyTR1 was also ~10,000-fold less potent with hCD3KI mouse T cells compared with human T cells, yet it robustly activated human CD3 knockin mouse T cells at concentrations less than required for cancer killing (Figures S4M and S4N). Thus, mouse T cells are markedly less responsive to GlyTR and cannot provide a meaningful assessment of GlyTR activity.

### GlyTR1 resists immunosuppressive mechanisms in the TME

**Figure 5.**
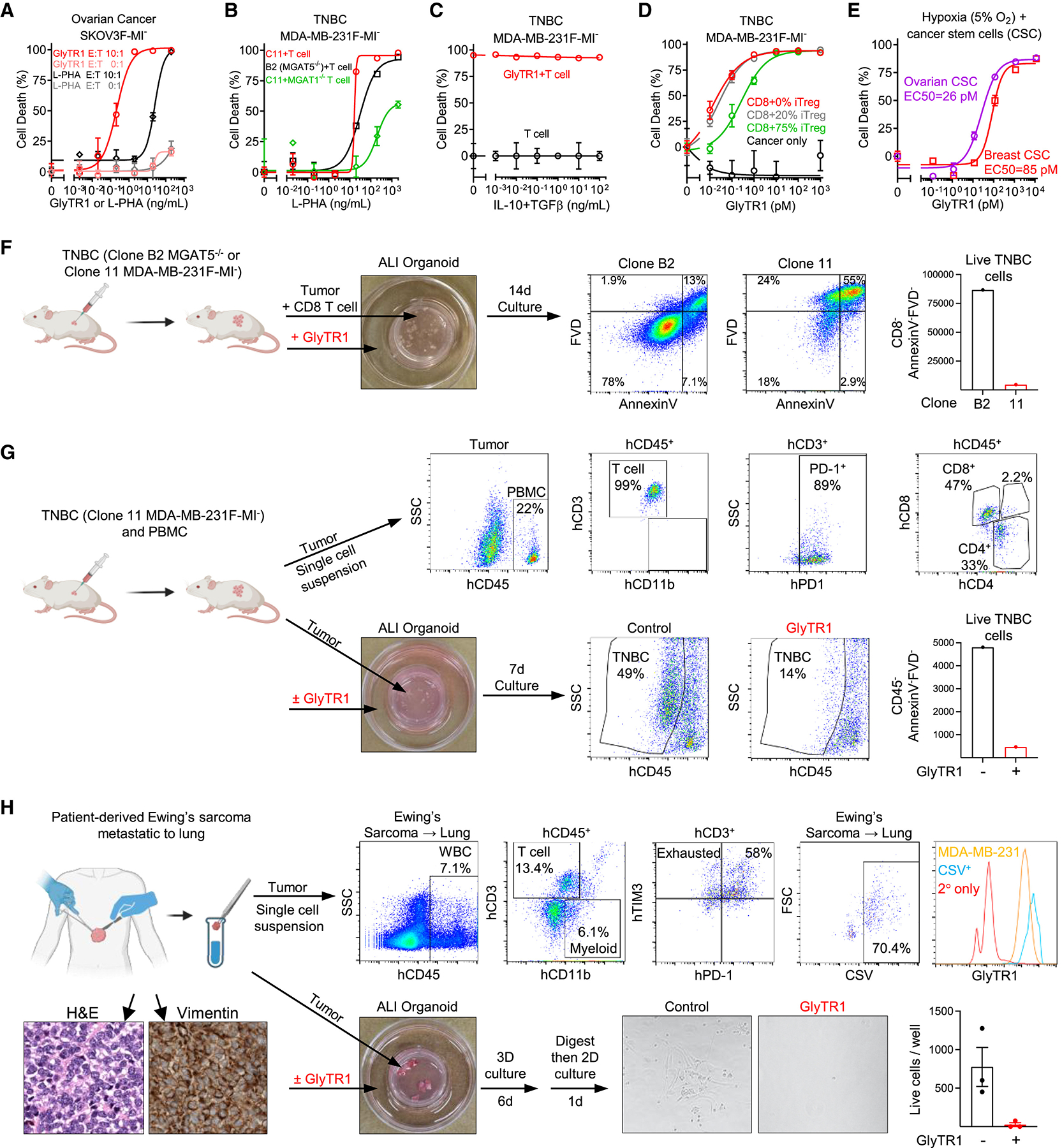
GlyTR1 overcomes multiple immunosuppressive mechanisms in the tumor microenvironment (A–E) Flow cytometry of cancer cell death triggered by GlyTR1 or L-PHA following co-culture of the indicated cancer cells/stem cells with/without human CD8^+^ T cells under the indicated conditions for 3 (A–D) or 2 (E) days. Patient-derived CSCs (E) were CD133^+^OCT4^+^SSEA3/4^+^ and cultured in a hypoxic chamber (5% O2, 5% CO2, and 90% N2). Data are the mean ± SEM of 3 biological replicates. (F and G) The indicated solid tumors grown in NSG mice without (F) or with PBMC co-injection (G) were analyzed by flow cytometry for immune cells as indicated or embedded in an air-liquid interface (ALI) organoid culture system with (F) or without the addition of exogenous PBMC (G). Tumor fragments were embedded in a transwell with a porous bottom containing a type I-A collagen solid matrix exposed to air and placed in a 60 mm dish containing organoid culture media with or without 100 ng/mL (F) or 500 (G) ng/mL) GlyTR1. After 7–14 days of ALI culture, single-cell suspensions were analyzed for live tumor cells by flow cytometry. FVD, fixable viability dye eFluor^™^ 780. (H) Fresh surgically resected vimentin^+^ Ewing’s sarcoma metastatic to the lung was analyzed by flow cytometry for hCD45^+^ immune cells or GlyTR1 binding to cell-surface-vimentin^+^ (CSV^+^) tumor cells, and the latter was compared with MDA-MB-231F-MI^−^ clone 11 TNBC. In parallel, an equal amount (by weight) of fresh tumor pieces was used in the ALI organoid system (as in G) and treated with or without GlyTR1 (500 ng/ml). After 6 days, the tumor was digested into single cells and plated/cultured overnight. Live adherent cells were imaged and counted. Flow cytometry confirmed adhered cells were CSV^+^. Data are the mean ± SEM of 3 biological replicates. See also Figure S5 and Table S1.

β1,6-branching is immunosuppressive when expressed in tumor cells^43,44,45^ as well as separately in B cells and T cells,^13,25,46,47,48,49^ the latter confirmed in a human clinical trial.^48^ This suggests that the binding of these glycans by the L-PHA domains in GlyTR1 may also function like a checkpoint inhibitor. To assess this, we examined whether the L-PHA lectin alone impacts allogeneic killing of cancer cells by MHC-mismatched T cells. Indeed, L-PHA significantly enhanced allogeneic killing by T cells, albeit at ~100-fold higher concentration than GlyTR1 (Figure 5A). Allogeneic killing triggered by L-PHA was reduced by lowering β1,6-branching both in tumor cells via *MGAT5* deletion (clone B2; Figure 5B) or in T cells via *MGAT1* deletion (Figure 5B). This confirmed that L-PHA impedes the immunosuppressive activity of β1,6-branching in both T cells and tumor cells. By contrast, GlyTR1 activity was enhanced rather than inhibited when β1,6-branching was reduced in primary T cells (Figures S1V and S1W). This is consistent with GlyTR1, but not L-PHA, having an anti-CD3 domain to trigger enhanced TCR clustering and signaling in the absence of β1,6-branching. Indeed, GlyTR2 activity, which also lacks L-PHA, was similarly enhanced by inhibiting β1,6-branching in primary T cells (Figure S5A). As the anti-CD3 domain in GlyTR1 primarily drives binding to T cells (Figures 1D and S1L), this will concentrate the attached L-PHA domain at the cell surface unlike L-PHA alone, thereby blocking β1,6 branching.

Interleukin (IL)-10 and TGFβ1 are potent immunosuppressive cytokines in the tumor microenvironment (TME) that inhibit cancer killing by bispecific antibodies,^50,51^ yet they did not reduce GlyTR1 killing activity individually or combined (Figures 5C and S5B). An immunosuppressive TME is also driven by regulatory T (Treg) cells. Cancer killing by GlyTR1 was not inhibited by the addition of 20% Tregs and only marginally reduced in the presence of 75% Tregs (Figures 5D and S5C). Hypoxia is another common immunosuppressive feature of the TME,^52^ yet hypoxia also had little impact on GlyTR1 killing of patient-derived CSCs that thrive in hypoxic niches (Figure 5E).

To confirm GlyTR1 can overcome an immunosuppressive syngeneic TME, we utilized the air-liquid interface (ALI) tumor organoid system that maintains the immune microenvironment.^53^ In this system, tumor pieces embedded in an air-exposed collagen matrix trans-well plate are placed in a larger plate containing organoid media (Figure 5F). To first explore GlyTR1 activity in this model, freshly isolated solid tumor fragments from wild-type (clone 11) and *MGAT5*-deleted (clone B2) MDA-MB-231F-MI^−^ TNBC (Figure 5F) solid tumors grown in NSG mice were co-embedded with CD8^+^ T cells in a collagen matrix and treated for 14 days with GlyTR1 added to the outer well. As in 2D culture (Figures 1I and 3D), GlyTR1 robustly killed wild-type (clone 11) but not *MGAT5*-deleted (clone B2) TNBC organoids (Figure 5F). To assess whether tumor-infiltrating T cells (TILs) are sufficient for GlyTR1 activity, TNBC (clone 11) tumors were grown in PBMC-humanized NSG mice (Figure 5G). Flow cytometry confirmed the tumor was infiltrated with ~22% T cells, of which 89% expressed PD-1 (Figure 5G). Despite this, GlyTR1 readily triggered endogenous TILs to kill the TNBC organoid cancer cells (Figure 5G).

To assess patient-derived tumors with syngeneic TILs and TME, we examined two fresh surgically resected metastatic solid tumors: Ewing’s sarcoma metastatic to lung and colon adenocarcinoma metastatic to liver (Figures 5H and S5D; Table S1). In both tumors, the majority of TILs were terminally exhausted (i.e., CD3^+^PD1^+^TIM3^+^) (Figures 5H and S5D). GlyTR1 target expression in cell-surface vimentin-positive (CSV^+^) Ewing sarcoma cells was significantly greater than in TNBC (clone 11) cells, and >95% were killed by GlyTR1 in the ALI organoid despite the terminally exhausted TILs (Figure 5H). In the metastatic colon tumor, GlyTR1 binding to EpCAM^Hi^CD45^−^ tumor cells was ~50% lower than TNBC (clone 11) cells (Figure S5D), a target density that should limit GlyTR1 activity. However, despite this and >70% terminally exhausted TILs, GlyTR1 killed >50% of ALI organoid tumor cells based on gating either EpCAM^Hi^CD45^−^ or CD45^−^ cells (Figure S5D). The latter were assessed as EpCAM expression is lost following epithelial-to-mesenchymal transition (EMT) in metastatic cells.^54^ Together, these data indicate that GlyTR1 overcomes multiple immunosuppressive mechanisms in the TME to trigger density-dependent cancer cell killing.

### GlyTR1 and GlyTR2 lack on-target, off-cancer toxicity

We performed immunohistochemistry (IHC) using L-PHA, GlyTR1, and GlyTR2 on two normal human TMAs to identify tissues at highest risk of on-target, off-cancer toxicity. Results were similar between L-PHA and GlyTR1, with low but variable staining of the brush border of the small bowel, surface epithelial cells of the stomach (intracellular), exocrine pancreas (acinar cells and intracellular), kidney cortex (glomerulus and tubules), and the molecular layer of the cerebellum (Figures S6A–S6E). Kidney staining is consistent with published data reporting L-PHA binding to glomerular podocytes (Bowman’s capsule), the brush border of the proximal tubule, and the thick portion of Henle’s loop.^55^ Higher concentrations revealed lower and variable staining in the adrenal gland, parotid duct, thyroid colloid, testis, prostate, uterus, spleen, and CNS white matter. Similar data were obtained for GlyTR2, with the highest and largely intracellular staining in mucin-producing tissues (esophagus, stomach, small and large bowel, endometrium, and salivary gland), kidney tubules, basal skin epidermis, and some cells in the pituitary (Figures S6F and S6G). Mucins are heavily O-glycosylated, and therefore the Tn antigen is normally expressed at high levels intracellularly in mucin-producing tissue. The secreted pituitary glycoprotein luteinizing hormone (LH) contains 4-sulfated-LacDiNAc.^36^ Although occupancy at the 4-position of GalNAc should prevent CD301 binding,^32^ intracellular generation of LacDiNAc prior to 4-O-sulfation is consistent with intracellular staining of GlyTR2 in some pituitary cells.

**Figure 6.**
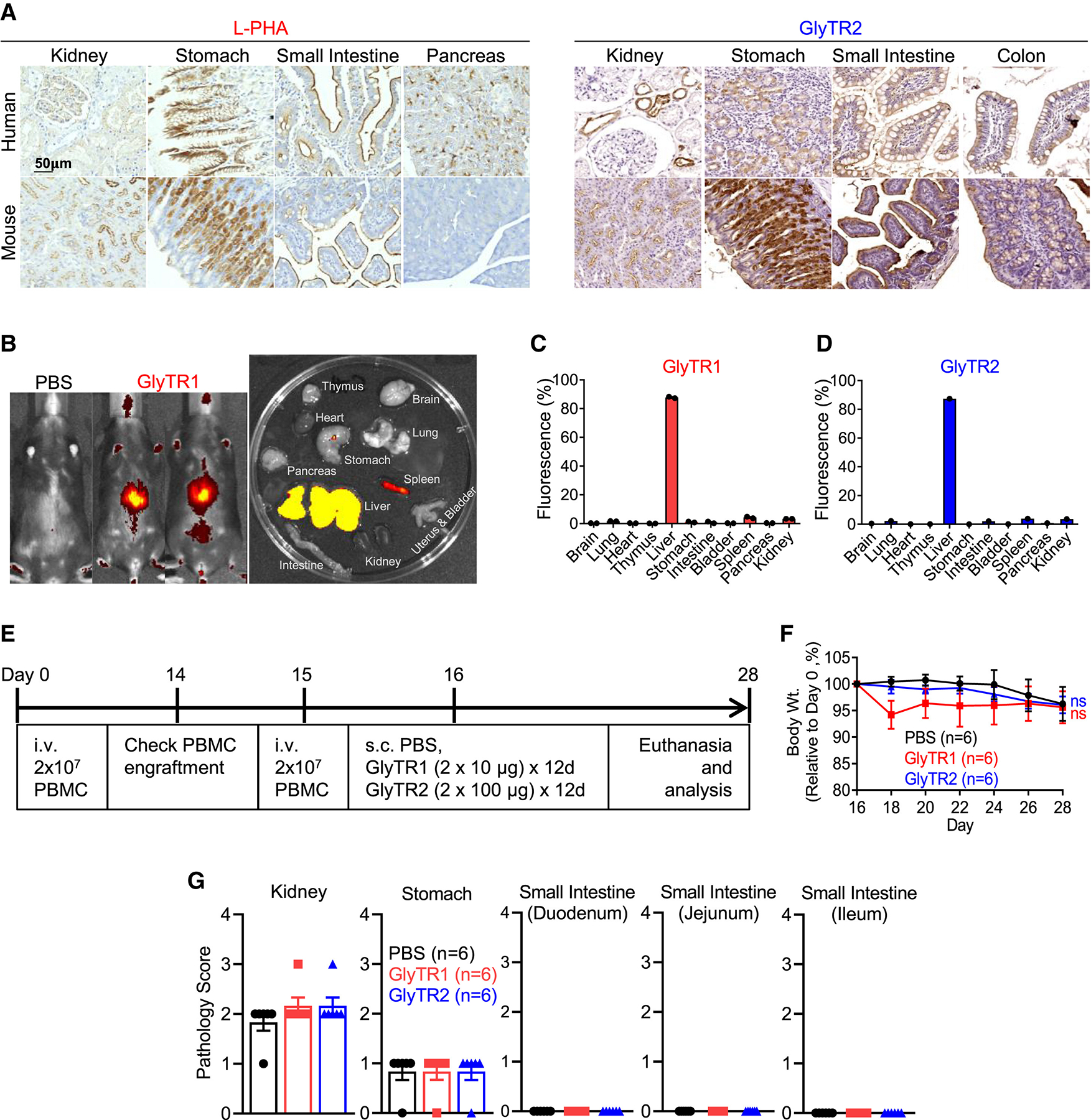
GlyTR1 and GlyTR2 are not toxic to mouse organs with human-like target density (A) IHC of the four highest staining human tissues along with their mouse counterparts is shown from TMAs containing 32 human (*n* = 3) and 22 mouse (*n* = 2–3) normal tissues stained with L-PHA-biotin or GlyTR2. (B–D) Biodistribution of GlyTR1 and GlyTR2 in the whole body and excised organs of C57BL/6 mice 8 h after injection of PBS (*n* = 1) or VivoTag680XL-labeled GlyTR1 (*n* = 2) or GlyTR2 (*n* = 1) via tail vein (B). Organ fluorescence is plotted as a percent of total fluorescence of all imaged organs after background subtraction of vehicle-injected mice (C and D). Each dot represents one mouse. Data represent the mean ± SEM. (E–G) NSG mice deficient for MHC class I and class II (NSG-MI/II^−^) engrafted with human PBMCs on days 0 and 15 were injected subcutaneously twice daily with PBS (*n* = 6), 10 μg GlyTR1 (*n* = 6), or 100 μg GlyTR2 (*n* = 6) for 12 days, followed by euthanasia and analysis on day 28. Microscopic changes in the indicated organs were graded blindly by IDEXX BioAnalytics as to pathological severity utilizing a standard grading system with 0 = no significant change, 1 = minimal, 2 = mild, 3 = moderate, and 4 = severe as per the International Harmonization of Nomenclature and Diagnostic (INHAND) Criteria standards (G). Each symbol represents one mouse. Other data are mean ± SEM (F and G). *p* values by nonparametric (Kruskal-Wallis) test corrected for Dunn’s multiple comparisons test for the last day (F). See also Table 1, Figures S6 and S7, and Tables S2 and S3.

To assess whether mice can model on-target, off-cancer toxicity risk, we co-stained mouse and human TMAs. Mice paralleled human expression in most tissue types except for mouse but not human pancreatic acinar cells being negative for intracellular L-PHA staining (Figure 6A). Lower-level staining in mice was also observed in the molecular layer of the cerebellum and spleen (data not shown).

To examine whether GlyTR proteins accumulate in normal tissues with the highest target expression, we injected fluorescently tagged GlyTR1 and GlyTR2 i.v. in mice. Neither GlyTR protein significantly accumulated in target-positive tissues (Figures 6B and S7A). Rather, both accumulated in the liver, with much smaller amounts in the spleen > bladder/kidney (Figures 6C and 6D). The former is consistent with the normal rapid liver uptake of proteins lacking a neonatal Fc receptor-binding domain.

Next, we performed a toxicity experiment in PBMC-humanized NSG-MI/II^−^ mice, which lack both MHC class I and II to minimize GvHD. 2 × 10^7^ human PBMCs were engrafted on days 0 and 15, with confirmation at day 14 (Figures 6E and S7B). On day 16, the PBMC-humanized mice were treated with PBS, GlyTR1, or GlyTR2 at doses that readily induced tumor regression *in vivo* (Figure 6E). Neither GlyTR protein significantly altered weight (Figure 6F), and no clinical toxicity was observed. One treated mouse developed mild head alopecia, which appeared grooming related. Clinical laboratory testing on day 28 revealed no treatment-induced differences in liver function, kidney function, electrolytes, pancreatic function, thyroid function, cholesterol, muscle (creatine phospho-kinase [CPK]), RBC, hematocrit, WBC, WBC differential, or platelets (Table 1). A small increase in glucose and decrease in hemoglobin were observed with GlyTR2, with the latter within the normal range for NSG mice^56^ (Table 1). A blinded histopathology analysis of three of the highest target-expressing tissues (kidney, stomach, and small intestine) by an independent veterinary pathologist found no treatment-related damage/inflammation (Figure 6G). Mononuclear infiltrates were observed perivascularly in the kidneys and within the serosa, muscular layer, and deep mucosa of the stomach in all groups, but no treatment-related findings were identified (Figure 6G). Analysis of the spleen revealed a small increase in size and total number of CD45^+^ human leukocytes in the GlyTR1 but not the GlyTR2 group (Table 1). However, neither treatment triggered differences in the percentage of human CD45^+^ leukocytes, CD4^+^ T cells, CD8^+^ T cells, CD19^+^ B cells, or Treg cells (Table 1). There was also no treatment-induced difference in the T cell activation markers CD69, CD25, or PD-1 or serum hIL-6 and hIFNγ, indicating treatment did not induce T cell activation despite target-positive tissue (Table 1).

The mice used above lacked MHC, which provides survival signals to T cells via basal TCR signaling. Indeed, human T cells in NSG-MI/II^−^ mice decline over time (e.g., from ~30% at 3 weeks to ~6% at 6 weeks). Coupled with the lack of upregulation of serum hIFNγ or T cell activation markers by GlyTR1, this suggests that the larger spleen in GlyTR1-treated NSG-MI/II^−^ mice is secondary to GlyTR1 providing survival signals rather than T cell activation/expansion. To confirm this, we repeated the toxicity experiment using NSG mice humanized with CD34^+^ hematopoietic stem cells that develop into functional human CD4^+^ and CD8^+^ T cells that recognize mouse MHC as self and therefore do not lack survival signals as with NSG-MI/II^−^ mice (Figure S7C).^57^ Indeed, GlyTR1 did not induce clinical toxicity (Table S2) nor alter weight (Figure S7D); spleen size/cellularity; the number or percentage of human CD45^+^ leukocytes; CD4^+^ and CD8^+^ T cells; CD19^+^ B cells; Treg cells; T cells positive for CD69, CD25, or PD-1; hIFNγ and hIL-6 levels; or major organ function (Table S2). By contrast, GlyTR1 readily expanded splenic human T cells in the presence of leukemic cancer cells (Figure S4G). Thus, GlyTR1 does not induce non-specific T cell expansion, but in the absence of MHC tickles TCR to promote survival.

### Safety of GlyTR lectins in humans

From 1963 to 1967, 14 case reports described the open-label use of i.v. PHA-P in aplastic anemia.^43,44,45,58,59,60,61,62,63,64,65,66,67,68^ PHA-P is a crude extract of red kidney beans that contains five tetrameric isolectins, with L-PHA at ~25% and the L subunit in another 25%.^69^ 47 patients were treated i.v. with bolus doses of 50 mg/day × 7–21 days or 250 mg over 50 h (Table S3). Doses up to ~2 times the mg/kg/day dose were used in our mouse experiments. Only mild side effects were reported in 10 of the 47 patients (Table S3). In contrast to i.v. PHA-P from red kidney beans, oral intake of uncooked red kidney beans leads to severe GI symptoms. This is consistent with positive L-PHA staining on the luminal brush border of the small intestine. As i.v. PHA-P did not induce GI symptoms in humans, i.v. GlyTR1 will also unlikely access the bowel lumen. Indeed, GlyTR1 did not accumulate in the stomach or intestine of mice (Figures 6B and 6C) despite human-like target expression (Figure 6A).

For GlyTR2, the CD301 lectin used is normally expressed in human DCs and macrophages, where engagement of multimeric CD301 by high-density Tn antigen triggers DC maturation and robust CD8^+^ T cell responses.^40^ The lack of chronic DC activation and associated CD8 T cell activation in normal humans indicates that normal human tissues lack sufficient target density to trigger CD301 binding/activation.

GlyTR1 and GlyTR2 are also unlikely to be highly immunogenic in humans. The L-PHA family of lectins is found in legumes (beans) and is routinely ingested by humans, suggesting pre-existing oral tolerance in the human population. Indeed, i.v. PHA-P did not induce acute hypersensitivity (Table S3).^43,44,45,58,59,60,61,62,63,64,65,66,67,68^ L-PHA is also poorly immunogenic in mice,^70^ and GlyTR1 did not trigger anti-GlyTR1 immunoglobulin M (IgM) or IgG antibodies significantly above background/detection limit (0.5 ng/ml) in C57BL/6 mice (Figures S7E–S7G), a level significantly below the 100 ng/mL typically required to impact drug activity.^71,72^ As CD301 used in GlyTR2 is a native human protein, GlyTR2 should be minimally immunogenic in humans. No anti-GlyTR2 IgM antibodies were induced in C57BL/6 mice (Figures S7E and S7H), but anti-GlyTR2 IgG antibodies were observed (Figure S7I). Without an IgM response, pre-existing class-switched IgG antibodies from memory B cells may have been triggered by GlyTR2. As human CD301 is a foreign protein in mice, this result has little relevance to the immunogenicity of GlyTR2 in humans. The half-life of GlyTR1 and GlyTR2 in NSG mice was 4.5 and 17.9 h, respectively (Figures S7J and S7K).

## Discussion

Development of antibody-based bispecific proteins and/or CAR cell immunotherapies has been restricted by the necessity of targeting cancer-specific cell-surface antigens to avoid on-target, off-cancer toxicity to normal tissue. Immunosuppression in the TME of solid cancers has also greatly hampered development. Glycans provide extremely high-density cell-surface targets in a wide assortment of cancers that can also be potently immunosuppressive, but most pure glycans are poorly targetable by antibodies. Here, we used lectins to generate two different pan-cancer immunotherapeutic bispecific proteins that distinguish high-target-density cancer cells from low-target-density normal tissue through multi-valency and high-avidity velcro-like binding to pure glycan antigens. The two GlyTR bispecific proteins trigger T cell-dependent killing of all tested cancer types with high target density, yet do not trigger on-target, off-cancer toxicity in mouse tissues with human-like target expression. Moreover, the GlyTR1 protein, via binding to immunosuppressive β1,6-branching, overcomes multiple immunosuppressive mechanisms in the TME, including T cell exhaustion, Treg cells, IL-10 + TGFβ1 cytokines, and hypoxia. Our approach allows a single off-the-shelf agent to potentially treat the vast majority of cancer types, including those with an immunosuppressive TME, while also providing improved safety by ignoring normal tissue with low target expression. GlyTR also did not trigger cytokine release syndrome in PBMC-humanized mice.

Our cancer models focused on metastatic disease rather than primary tumors, as both GlyTR targets are major drivers of tumor progression and metastasis, resulting in target density (and associated sensitivity to killing) being highest in late-stage metastatic disease. This patient group also has the highest unmet clinical need and will be the population targeted in initial clinical trials.

Defining the threshold density of target expression for robust killing and safety of GlyTR proteins *in vivo* in humans will require clinical studies. Our flow cytometry data suggest cell-surface expression that is >6–8 times that of resting CD4^+^ T cells are potently killed by GlyTR proteins while sparing low-density cell types. However, liquid cancer cells appear more sensitive than solid cancer cells at similar target densities, possibly because of T cell help by the former and/or PD-L1 in the latter. As IHC is poorly quantitative, immunofluorescence microscopy and/or flow cytometry will best inform future clinical studies.

GlyTR1 was considerably more potent than GlyTR2. This disparity may reflect absolute differences in target density, the specific proteins the glycans are attached to, the additional anti-CD3 domain present in GlyTR1 triggering more robust T cell activation, and/or GlyTR1 targeting immunosuppressive β1,6-branching. The latter inhibit T cell activity by interacting with galectins to form a macromolecular lattice that suppresses TCR clustering/signaling at the immune synapse,^25,26^ enhances surface retention of CTLA-4^13^ and PD-1,^49^ and inhibits T_H_1/T_H_17 differentiation while promoting Treg cells.^46^ By competing with galectins for binding to β1,6-branching, GlyTR1 appears to disrupt the galectin lattice to block these phenotypes. Similarly, disruption of the lattice in tumor cells will promote immune synapse formation with T cells.^43,44,45^

Like all antigens, downregulation of glycan targets would lead to resistance. However, as β1,6-branching and Tn antigen promote tumor growth and metastasis^12,13^ and are associated with poor prognosis, downregulation would be detrimental to the tumor and therefore may still promote survival even with loss of antigen. GlyTR2 also has an advantage over antibody-based targeting of Tn-MUC1^23^ or Siayl/Tn-TAG-72,^73^ as tumor escape will occur simply by addition or deletion of sialic acid, respectively, while GlyTR2 should remain effective due to binding both Tn and Siayl-Tn. As both GlyTR targets are co-expressed in most cancers, combination treatment should further limit tumor escape.

GlyTR safety is also likely promoted by tissue barriers that prevent access to the target-expressing luminal membranes of the stomach, small intestine, and kidney podocytes/tubules, the three highest GlyTR target-expressing normal tissues (Figure 6A). Consistent with this, GlyTR proteins do not accumulate in the stomach/small intestine or kidneys of mice (Figures 6B–6D). Moreover, oral but not i.v. L-PHA causes severe GI symptoms in humans, consistent with only the former accessing the luminal membranes of the stomach/bowel. The glomerular basement membrane has a filtration barrier to proteins >60 kDa, which should exclude GlyTR proteins (predicted MW ~182 kDa and ~104 kDa) from accessing podocytes/tubule cells. Similarly, the blood-brain barrier should exclude GlyTR proteins from the brain.

The GlyTR protein design should be readily applicable to generating GlyTR CAR cells that target high-density TACAs. There are numerous other lectins and associated TACA-binding partners that may be employed to make additional GlyTR bispecific proteins or CAR T cells. For example, high-density expression of poly-N-acetyllactosamine or sialyl-Lewis^x/a^ in cancer could be targeted with lectins.^1^ Different lectin domains could also be combined for dual/triple-TACA targeting.

## Limitations of the study

Although GlyTR1 *in vivo* activity was independently replicated at the National Cancer Institute, all of our mouse models lacked a syngeneic immunecompetent system. This was despite generating and assessing both a mouse reactive GlyTR1 and human CD3ε,δ,γ knockin mouse T cells, with both displaying a >10,000-fold reduction in activity. However, GlyTR1 readily killed patient-derived tumor organoids despite an intact syngeneic immunosuppressive TME that harbored terminally exhausted T cells. GlyTR1 also overcomes multiple immunosuppressive mechanisms and acts like a checkpoint inhibitor via binding to immunosuppressive β1,6-branching. Indeed, high i.v. doses of L-PHA in two melanoma patients resulted in inflammatory infiltrates and tumor regression.^59,74^ A second limitation is that our i.p. TNBC model (Figures 4F and 4G) does not reflect a typical metastatic site; however, we observed similar positive results in an i.v. lung metastatic TNBC model (Figure S4D).

Assessing GlyTR target density and killing of primary cells is limited by the need for growth factors in the culture to prevent death, many of which upregulate β1,6-branching via activation of the ERK/MAP kinase pathway. Normal tissue organoids are also problematic, as they similarly require multiple growth factors and lack barriers that would block GlyTR access *in vivo*. For example, kidney organoids do not possess a functional glomerular basement membrane/filtration barrier.^75^ Cerebral organoids similarly lack a blood-brain barrier. Our IHC data assessed unstimulated primary cells but utilizes non-linear signal amplification and is semi-quantitative. By contrast, GlyTR immunofluorescence is quantitative and displayed little binding to multiple normal tissues but increasing binding to colon cancer based on stage and metastasis (Figure 3C). Lack of GlyTR toxicity in mice with human-like target expression provides the most physiological analysis of safety, as cells are not artificially exposed to growth factors and tissue barriers are intact (Figure 6; Tables 1 and S2). Moreover, the lectin used in GlyTR1 was safe when i.v. delivered at doses much higher than those required for GlyTR1 to kill cancer cells *in vivo* (Table S3), while the lectin used in GlyTR2 is a native human protein expressed in monocytes/DCs that is not activated *in vivo* by self antigens. Ultimately, human clinical trials are required to confirm the efficacy and safety of GlyTR therapeutics. In this regard, GlyTR1 is beginning GMP manufacturing and IND-enabling studies for a planned Phase 1 basket trial in refractory solid cancer as part of the “NCI Experimental Therapeutics” (NExT) program (https://next.cancer.gov). Combining GlyTR1 and GlyTR2 and/or developing other GlyTRs using different lectins should further expand the likelihood for successful application of GlyTR therapeutics in humans.

## Resource availability

### Lead contact

Requests for further information and resources should be directed to and will be fulfilled by the lead contact, Michael Demetriou (mdemetri@uci.edu).

### Materials availability

Cell lines newly generated in this work are available upon request to the lead contact. Availability of GlyTR reagents is limited and will require a short scientific proposal, a statement on potential commercial uses, and a completed materials transfer agreement with final approval by the lead contact.

### Data and code availability

Original microscopical images can be requested from the lead contact. This paper does not report original code. Any additional information required to reanalyze the data reported in this paper is available from the lead contact upon request.

## STAR★Methods

### Experimental model and study participant details

#### Human samples

No human subjects research was performed. Human cancer remnant tissues were collected by the Shared Tissue Resource at UCI from patients undergoing surgery at UCI Medical Center as part of a research tissue sample collection protocol approved by the Institutional Review Board (IRB) of the University of California, Irvine. Clinical data of this tumor samples are reported in Table S1. Random de-identified leukopaks and whole blood from healthy donors for isolation of primary T cells were obtained from outside vendors and/or the Institute for Clinical and Translational Science UCI, respectively.

#### Animals

Male and female NSG (NOD.Cg-Prkdc^scid^ Il2rg^tm1Wjl^/SzJ, #005557), NSG-MI/II^−^ (NOD.Cg-*Prkdc*^*scid*^ *H2-K1*^*b-tm1Bpe*^ *H2-Ab1*^*g7-em1Mvw*^ *H2-D1*^*b-tm1Bpe*^ *Il2rg*^*tm1Wjl*^/SzJ, #025216) and C57BL/6J (#000664) mice were obtained from The Jackson Laboratory and bred in-house. Female CD34^+^ Humanized NSG mice were obtained from The Jackson Laboratory. Mice were cared and housed in ventilated sterile-barrier cages in the animal facilities with automatic 12-hour light/dark cycles and *ad libitum* food and water at the University of California, Irvine. Animal experiments were approved by the Institutional Animal Care and Use Committee at the University of California, Irvine.

#### Cells

All established cancer cell lines were cultured at 37°C in 5% CO_2_ incubator with RPMI1640 media (Corning) supplemented with 10% heat-inactivated FBS (Sigma), Penicillin-Streptomycin-Glutamine (ThermoFisher) and 2-Mercaptoethanol (ThermoFisher). Patient-derived stage 4 CD133^+^OCT4^+^SSEA3/4^+^ ovarian, TNBC and lung cancer stem cells and respective serum-free expansion media were purchased from Celprogen Inc. and cultured at 37°C in humidified incubator in hypoxic chamber (Stemcell Technologies) with 5% O_2_, 5% CO_2_ and 90% N_2_. Cell lines were authenticated for target expression but cell origin. Patient-derived cancer cells were cultured in complete RPMI1640 media. Normal human renal, renal proximal tubule, pulmonary alveolar, prostate and colonic epithelial cells and hepatocytes and brain vascular adventitial fibroblast (ScienCell) were cultured in their respective media from the vendor. Complete media for the normal human cells contain essential and non-essential amino acids, vitamins, organic and inorganic compounds, hormones, growth factors, trace minerals and 2–5% FBS. ExpiCHO-S cells were purchased and maintained in ExpiCHO^™^ Expression Medium (ThermoFisher) at 37°C in humidified incubator with 5% CO_2_. Deletion of *Gale* and *Mgat1* in ExpiCHO-S cells or *MGAT1* and/or *TRAC* in primary human T cells or *B2M* in Capan1F cells were performed using CRISPR-Cas9 editing. A clone of Gale^−/−^ ExpiCHO-S cells were selected and expanded from limiting dilution. Mgat1^−/−^ ExpiCHO-S cell pool was enriched by negatively selecting for cells deficient of β1,6GlcNAc-branched N-glycans using biotinylated L-PHA (Vector Laboratories) and Streptavidin RapidSpheres (Stemcell Technologies). Deletion of *MGAT5*, *COSMC* and/or *B2M* in MDA-MB-231F or SKOV3F were performed as a service by Synthego. MDA-MB-231F (clone 2, 4, 5, 11, 12 and B2) were selected and expanded by limiting dilution from enriched cell pools collected from sorting with BD FACSAria Fusion Sorter. Insertion of firefly luciferase was done using lentiviral transfection. Mycoplasma was regularly tested with MycoAlert^™^ mycoplasma testing kit (Lonza).

### Method details

#### Generation of GlyTR proteins

GlyTR genes were synthesized and subcloned into pcDNA3.1+ plasmid vector (Genscript). GlyTR proteins were produced by transient transfection using Mgat1^−/−^ ExpiCHO-S for GlyTR1 or unmodified ExpiCHO-S cells for GlyTR2. GlyTR proteins were purified by 6xhistidine-tag-specific affinity chromatography followed by size exclusion chromatography using AKTA systems (Cytiva). Protein purity was estimated by immunoblot with HRP-conjugated anti-6xHis (Abcam) and comparing the differential abundance of GlyTR proteins loaded at 100%, 5% and 1% dilutions in reducing SDS-PAGE for silver staining (ThermoFisher). Protein concentrations were determined using BCA assay (ThermoFisher) and were stored in aliquots at −80°C or −20°C for short-term. VivoTag680XL-labeled GlyTR proteins were generated using the VivoTag 680XL protein labeling kit (PerkinElmer). Alexa Fluor 647-labeled (for flow cytometry) and FITC-labeled GlyTR1 for immunofluorescence were generated using protein labeling kit (ThermoFisher).

#### PBMC and regulatory T cell preparation

PBMCs and CD8^+^ T cells are isolated by negative selection from Leukopaks (Stemcell Technologies) or whole blood (Institute for Clinical and Translational Science program or ICTS at UCI) using the Direct human PBMC isolation kit and/or human CD8^+^ T cell enrichment kit (Stemcell Technologies) per manufacturer’s protocols. Regulatory T cells (Tregs) were induced with negatively isolated human naïve CD4^+^ T cells (Stemcell Technologies) with anti-CD3ε/CD28 antibodies (1 and 2 μg/mL, respectively), IL-2 (100 U/mL) and TGFβ1 (2 ng/mL). Percent of induced regulatory T cells were confirmed with flow cytometric assessment of FOXP3 staining using the Foxp3/Transcription Factor Staining Buffer Set (ThermoFisher).

#### Flow cytometry

Anti-human antibodies to CD3 (OKT3), CD3 (UCH1), CD4 (OKT4), CD8a (SK1), CD19 (HIB19), CD25 (BC96), CD45 (HI30), CD69 (FN50), HLA-A,B,C (W6/32), PD-1 (EH12.2H7) and anti-mouse antibodies to CD3ε (17A2), CD4 (RM4-5), CD45 (30-F11) were purchased from Biolegend. Antibodies to CD3 (OKT3), CD28 (CD28.2), FOXP3 (236A/E7), B220 (RA3-6B2), CD8a (53-6.7), CD19 (1D3) and Fixable Viability Dye eFluor^™^ 780 (FVD) were from ThermoFisher. DyLight488 anti-6xHis (AD1.1.10) and polyclonal HRP anti-6xHis were from Abcam. L-PHA-Fluorescein, biotinylated-L-PHA, Concanavalin A-Fluorescein and Streptavidin-DyLight649 were from Vector Laboratories; CD301-6xHis was from R&D Systems. GlyTR cell surface binding experiments were performed with 30-minute incubation on ice followed with additional 30-minute incubation with secondary antibody DyLight488 anti-6xHis on ice in the dark using primary human lymphocytes and cancer cells pre-treated with or without kifunensine or Tn-, GalNAc- or GlcNAc-supplemented staining buffer (1% Bovine serum albumin (BSA) and 0.1% Sodium Azide in 1x PBS for GlyTR1 or 1% BSA, 0.14M NaCl and 2.5mM CaCl_2_ in 10 mM HEPES (pH 7.4) for GlyTR2). Flow cytometry was performed using an Attune NxT (ThermoFisher), Novocyte 3000 (ACEA), BD LSRFortessa (BD Biosciences), MACSQuant Analyzer 10 or MACSQuant Analyzer 16 (Miltenyi).

#### *In vitro* co-culture assay

CFSE-labeled cancer cells were co-cultured with PBMCs or enriched CD8^+^ T cells supplemented with or without cytokines or induced regulatory T cells on 96-well plates in tissue culture incubator with 5% CO_2_ at 37°C. Co-culture of ovarian, TNBC and lung cancer stem cells were done in a hypoxic chamber with 5% O_2_, 5% CO_2_ and 90% N_2_. After co-culture, samples were then stained with Fixable Viability Dye eFluor^™^ 780 (FVD) for flow cytometric analysis. Cell death was calculated using equation: Cancer Cell Death % = 100 – (live cell # treated with GlyTR ÷ live cell # without GlyTR) × 100 where live CFSE^+^FVD^−^ cancer cells were gated for analysis.

#### GlyTR protein accumulation with tumor in mice

NSG mice were engrafted intravenously via tail vein with/without breast cancer cell line deficient of MHC-I and COSMC (MDA-MB-231F-MI^−^C^−^). Tumors were allowed to establish for 19 or 35 days. Lung tissues were excised and imaged for luminescence (tumor) and fluorescence (GlyTR1 or GlyTR2) from mice 2 hours after i.v. injection with VivoTag680XL-labeled GlyTR1/GlyTR2 and 10–20 minutes post D-luciferin i.p injection.

#### Biodistribution of GlyTR proteins in C57BL/6 mice

C57BL/6 mice were i.v. injected with PBS, GlyTR1 or GlyTR2 labeled with VivoTag680XL. At various time points, fluorescent imaging of whole body (live) and excised organs were performed. Fluorescence of each organ was quantified as a percent of total fluorescence of all imaged organs after background subtraction of PBS injected mice. Luminescent and fluorescent signals were detected with Xenogen IVIS imager.

#### Tumor regression in humanized mice

NSG mice were injected i.p. with pancreatic cell line (Capan1F-MI^−^), breast cancer cell lines (MDA-MB-231F-MI^−^ or MDA-MB-231F-MI^−^C^−^) or ovarian cancer cell line (SKOV3F-MI^−^). Following tumor establishment, the mice were then engrafted i.p. with 1×10^7^ purified primary human CD8^+^ T cells (1^st^ injection) or followed by repeated engraftment of CD8^+^ T cells (1×10^7^ cells for MDA-MB-231F-MI^−^ and SKOV3F-MI^−^ models or 2×10^6^ for MDA-MB-231F-MI^−^C^−^ model) every 3–4 days. PBS or GlyTR treatment via i.p. injection started after the first CD8^+^ T cell engraftment and continued twice daily. NSG-MI/II^−^ mice deficient for MHC class I and class II were inoculated i.v. with 2×10^7^ human PBMCs and 0.5×10^6^ breast cancer cells (MDA-MB-231F-MI^−^C^−^), then starting on day 1 treated with/without GlyTR1 twice daily subcutaneously for 2 weeks. Tumor burden was monitored with bioluminescent imaging using Spectral Imaging Instrument AMI-HT or Xenogen IVIS imager. For leukemic models, NSG mice were inoculated i.v. with T cell leukemia (TCRβ^−/−^ Jurkat) cells via tail vein injection. Following tumor expansion for 14–15 days, the mice were engrafted i.v. with 2×10^7^ PBMCs and followed with daily i.v. injection of GlyTR1 or GlyTR2^CD301(3)xCD3^. Tumor burden was examined by flow cytometric analysis of splenocytes.

#### Generation of C57BL/6^hCD3ε,δ,γ^ mice

A transgenic mouse with T cells that express only the human CD3 complex (ie C57BL/6^hCD3ε,δ,γ^) was generated by the UC Irvine transgenic mouse facility. A custom modified BAC clone with human genomic DNA containing the three *CD3EDG* genes flanked by mouse genomic DNA 5’ and 3’ of the mouse CD3ε,δ,γ genomic region was generated by GeneBridges (Germany). This BAC clone and homology-dependent repair (HDR) by CRISPR-Cas9 was used to replace the mouse CD3 complex (i.e. CD3ε, δ, and γ) with human CD3ε, δ, and γ in mouse embryonic stem (ES) cells. Multiple ES cell clones were correctly targeted based on Southern analysis of both the 5’ and 3’ ends of the targeting construct. A clone with high euploidy and single copies of the mouse and human CD3 gene clusters were utilized for IVF to successfully generate chimeric animals that was used to generate mice heterozygous and then homozygous mice for the human CD3 gene cluster. Flow cytometry was used to confirm absence of mouse CD3 and presence of human CD3 in homozygous mice.

#### Organoid cultures

Air-liquid interface (ALI) organoid plates were prepared as described.^53^ For organoid culture from subcutaneous mouse tumors, wildtype (clone 11) or *MGAT5*-deleted (clone B2) MDA-MB-231F-MI^−^ cells were inoculated and allowed to grow in NSG or PBMC-humanized NSG mice. Resected tumor tissues were minced on ice into fine pieces and mixed with 1 mL of cell matrix type I-A collagen. Tumor tissues were weighed and kept similar between treatment and control group prior to mixing with 1 mL collagen before adding into the ALI system with/without adding CD8^+^ T cells and/or GlyTR1. After co-culture, media was removed from the ALI organoid plates, and organoids were dissociated from collagen matrix with 200 units/mL of collagenase at 37°C for 15 minutes. Samples were washed using ADMEM/F12 media and digested in Liberase-TL for 15 minutes at 37°C. Samples were washed twice in ADMEM/F12, triturated with a P1000 pipet and passed over a 70-micron strainer. Single cells were then used for flow cytometric analysis. Patient derived organoid cultures were done using the method similar to as described above. The ALI collagen gel matrices were prepared by mixing cell matrix type I-A collagen with media (50 μl of 20x concentrated Ham’s F12 culture media with 50 μL of L-WRN condition media) and reconstitution buffer (2.2 g NaHCO_3_ in 100 mL of 0.05 N NaOH and 200 mM HEPES) at the ratio of 8:1:1 under the sterile condition on ice. De-identified fresh remnant human tumor tissues were obtained through Experimental Tissue Shared Resource at UCI from patients undergoing surgery at UCI Medical Center. Tumor tissues were then minced and added in the ALI systems after mixing with 1 mL of collagen gel matrices. The organoids were cultured with/without GlyTR1 before harvesting for single cell suspension preparation. Single cell suspension was then seeded into a 96-well tissue culture-treated plate to allow cancer cell to adhere to the surface. Debris and non-adherent immune cells were removed 24-hour by repeated gentle washing using 1x PBS. The wells on the 96-well plate were then imaged using microscope before trypsinization and counting.

#### Immunofluorescence and Immunohistochemistry

Human colorectal carcinoma progression tissue microarray (CHTN-CRC2, Cooperative Human Tissue Network, University of Virginia) was stained with GlyTR1-FITC at 5 μg/mL before analysis by immunofluorescent microscopy (Aperio Versa 200, Model DM6 B, Leica). Human tissue microarrays from US Biomax (FDA999u, FDA999w and BR1005b), US Biolabs (FDA999-1), Novus Biologicals (NBP2-30169) and mouse (C57BL/6) tissue microarray from Pantomics (AMS545) were stained with L-PHA-biotin (0.25 μg/mL for FDA999u), GlyTR1 (0.5 μg/mL for FDA999w or 0.67 μg/mL for FDA999-1) or GlyTR2 (3 μg/mL for FDA999-1 and BR1005b) with detection by streptavidin-HRP (Biolegend) or anti-6xHis-HRP (Abcam).

#### Toxicity in humanized mice

NSG-MI/II^−^ mice were engrafted i.v. with 2×10^7^ human PBMCs on each of day 0 and 15. Retro-orbital blood sampling was performed and analyzed by flow cytometry on day 14. Mice (n=6 animals per group) were then injected subcutaneously twice daily with PBS, GlyTR1 or GlyTR2 for 12 days before euthanasia for analysis on day 28. For clinical chemistry, blood was pooled equally from 2 mice of the same treatment group to ensure sufficient volume for analysis; therefore, mean±SEM represents 6 mice (3 pooled blood samples). For TSH, 0.03 data points represent <0.03 (detection limit of the assay).

Complete blood count was performed for each mouse (n=6 per group). Similarly, CD34^+^ humanized NSG mice (HuNSG from Jackson Laboratory) were injected subcutaneously twice daily with PBS, 2.5μg, 5μg or 10μg GlyTR1 for 10 days before euthanasia for analysis. Blood was pooled equally from 3 mice of the same treatment group (1 pooled blood sample per group) to ensure sufficient volume for analysis by Antech Diagnostics. Complete blood count was analyzed for each mouse by a Scil Vet abc Animal Blood Counter. Splenocytes were prepared and analyzed by flow cytometry. Plasma was prepared from whole blood and analyzed with Enzyme-linked immunosorbent assay (ELISA).

#### ELISA, Immunogenicity and half-life in mice

Cytokines (IFNγ, IL-10 or IL-17A) from co-culture supernatants were measured with sandwich ELISA assay using anti-IFNγ (BD Biosciences), IL-10 or IL-17A (Biolegend) as capture antibodies and biotinylated detection antibodies followed by streptavidin-HRP for detection. Absorbance was measured at 450 nm. GlyTR1 or GlyTR2 (10 μg/ml) coated plates were incubated for 2 hours with serially diluted serum from C57BL/6 mice treated with vehicle, GlyTR1 or GlyTR2 i.v. daily for 7 days, boosted at day 14 with vehicle, GlyTR1 or GlyTR2 i.v. and then bled on day 21. After washing, bound IgM or IgG antibodies were detected by anti-mouse IgM-HRP and IgG-HRP, respectively. Presented data are background subtracted.

#### Quantification and Statistical Analysis

No statistical method was used to pre-determine sample size, but was based on previous work using similar models. The experiments were not randomized. In xenotransplant tumor models, mice were evenly split based on tumor burden before treatment; all others were randomized based on sex and age. Comparison of *in vitro* dose dependent and *in vivo* time-dependent tumor killing were analyzed by non-linear and linear regression, respectively. All other data were analyzed by parametric tests (Student’s t-test or repeated measure one-way/two-way ANOVA with Sidak’s multiple comparisons test) when data normality was confirmed by the Shaprio-Wilk test; otherwise, nonparametric tests (Kruskal-Wallis with Dunn’s multiple comparisons test) were employed. The specific statistical test used is specified in the Figure legend. n represents number of biological replicates or mice depending on the experiment. Data are presented as the mean ± standard error of the mean as indicated in the Figure legends. No data outliers were excluded. The investigators were blinded for histopathology analysis of GlyTR treated mice; other experiments were unblinded. *In vivo* activity of GlyTR1 was independently confirmed by scientists at the National Cancer Institute. Computational analysis was performed with GraphPad Prism.

## Supplementary Material

Sup Tables 1 - 3

Supplementary_Figures

Download all supplementary files included with this article

What’s this?

Download: Download Acrobat PDF file (366KB)

Document S1. Tables S1–S3.

Download: Download Acrobat PDF file (32MB)

Data S1. Primary image for GlyTR1 immunofluorescence staining of a human colorectal carcinoma progression TMA, related to Figure 3. Immunofluorescence staining of GlyTR1-FITC on a human colorectal carcinoma progression tissue microarray (CHTN-CRC2 from University of Virginia). The tissue microarray contains 13 tissues with replicates up to 8 different individuals. Control tissues: Con1 - spleen, Con2 - kidney, Con3 - placenta, Con4 - liver. Bnc - normal non-neoplastic colonic mucosa from non-cancer cases, Bc - normal non-neoplastic colonic mucosa from cancer cases, Bi - inflamed and/or regenerative non-neoplastic mucosa (ulcerative colitis), AS - adenomas < 2 cm in maximum dimension, AL - adenomas > 2 cm in maximum dimension, CE - primary invasive adenocarcinoma, pathologic stage T1 or T2, CL - primary invasive adenocarcinoma, pathologic stage T3 or T4, LN - colorectal adenocarcinoma metastatic to lymph nodes, same cases as CL primary cancers, M - colorectal adenocarcinoma metastatic to distant sites.

## Figures and Tables

**Table 1.** Toxicity assessment of GlyTR1 and GlyTR2 in PBMC-humanized NSG-MI/II^−^ mice

Empty Cell	PBS (*n* = 6) (mean ± SEM)	GlyTR1 (*n* = 6) (mean ± SEM)	*p* value^a^	GlyTR2 (*n* = 6) (mean ± SEM)	*p* value^a^
**Chemistry**					
Total protein (g/dL)	5.9 ± 0.2	5.8 ± 0.2	ns	5.5 ± 0.3	ns
Albumin (g/dL)	2.8 ± 0.2	2.7 ± 0.2	ns	2.9 ± 0.1	ns
Globulin (g/dL)	3.1 ± 0.2	3.1 ± 0.1	ns	2.6 ± 0.2	ns
AST (IU/L)	492 ± 119	454 ± 161	ns	297 ± 35	ns
ALT (IU/L)	264 ± 100	260 ± 162	ns	102 ± 20	ns
ALK Phos (IU/L)	91 ± 18	80 ± 10	ns	73 ± 1	ns
T. Bilirubin (mg/dL)	0.8 ± 0.5	0.4 ± 0.1	ns	0.2 ± 0.1	ns
BUN (mg/dL)	26.0 ± 1.0	25.0 ± 1.0	ns	28.3 ± 2.4	ns
Creatinine (mg/dL)	0.3 ± 0.0	0.3 ± 0.0	ns	0.3 ± 0.0	ns
Sodium (mEq/dL)	158.0 ± 1.0	161.0 ± 3.6	ns	155.3 ± 4.8	ns
Chloride (mEq/dL)	115.0 ± 1.0	119.0 ± 3.6	ns	118.0 ± 2.6	ns
Potassium (mEq/dL)	5.9 ± 0.3	5.6 ± 0.3	ns	5.8 ± 0.1	ns
Calcium (mg/dL)	10.3 ± 0.4	10.2 ± 0.3	ns	9.9 ± 0.1	ns
Phosphorus (mg/dL)	10.6 ± 0.5	10.3 ± 0.5	ns	10.3 ± 0.2	ns
Glucose (mg/dL)	86.0 ± 15.7	103.0 ± 11.8	ns	154.0 ± 9.5	0.0341
Amylase (IU/L)	499.0 ± 38.2	534.0 ± 21.0	ns	536.0 ± 10.2	ns
Precision PSL (IU/L)	25.0 ± 1.0	28.0 ± 2.6	ns	30.3 ± 1.8	ns
TSH (ng/mL)	0.04 ± 0.01	0.03 ± 0.00	ns	0.03 ± 0.00	ns
Creatine phospho-kinase (CPK) (IU/L)	865 ± 247	1,211 ± 367	ns	946.0 ± 34.4	ns
Cholesterol (mg/dL)	91.0 ± 19.1	62.0 ± 7.0	ns	70.0 ± 4.0	ns
**Complete blood count**					
RBC (× 10^6^/uL)	8.9 ± 0.7	8.4 ± 0.3	ns	7.1 ± 0.4	ns
Hemoglobin (g/dL)	15.9 ± 0.7	14.7 ± 0.3	ns	13.3 ± 0.5	0.0045
Hematocrit (%)	48.9 ± 4.4	46.8 ± 2.1	ns	40.0 ± 2.1	ns
WBC (× 10^3^/uL)	22.7 ± 10.7	29.7 ± 9.0	ns	11.0 ± 2.8	ns
Platelets (× 10^3^/uL)	748.2 ± 207.7	707.7 ± 227.4	ns	721.7 ± 146.6	ns
Lymphocytes (%)	38.8 ± 3.4	37.5 ± 2.5	ns	47.4 ± 2.7	ns
Monocytes (%)	14.6 ± 0.2	14.3 ± 0.7	ns	14.6 ± 0.3	ns
Granulocytes (%)	46.6 ± 3.3	48.2 ± 1.9	ns	38.1 ± 2.9	ns
**Spleen: flow cytometry**					
hCD45+ splenocytes (× 10^8^)	0.5 ± 0.1	1.5 ± 0.3	0.0463	0.2 ± 0.1	ns
hCD4^+^ (% of hCD45^+^)	23.4 ± 3.4^b^	23.8 ± 2.6	ns	23.7 ± 3.1	ns
hCD8^+^ (% of hCD45^+^)	47.3 ± 9.4^b^	40.7 ± 5.3	ns	46.4 ± 7.1	ns
hCD19^+^ (% of hCD45^+^)	15.9 ± 6.1	26.1 ± 4.4	ns	18.4 ± 4.6	ns
FOXP3^+^ (% of hCD4^+^)	0.3 ± 0.1	0.6 ± 0.2	ns	0.4 ± 0.0	ns
hCD69^+^ (% of hCD4^+^)	12.4 ± 2.0^b^	14.4 ± 1.7	ns	22.9 ± 4.3	ns
hCD69^+^ (% of hCD8^+^)	13.1 ± 1.9^b^	8.8 ± 0.9	ns	12.3 ± 2.2	ns
hCD25^+^ (% of hCD4^+^)	0.9 ± 0.3^b^	1.4 ± 0.3	ns	1.2 ± 0.4	ns
hCD25^+^ (% of hCD8^+^)	0.6 ± 0.1^b^	0.5 ± 0.2	ns	0.8 ± 0.2	ns
hPD1^+^ (% of hCD4^+^)	90.9 ± 1.5^b^	93.7 ± 0.4	ns	92.9 ± 0.9	ns
hPD1^+^ (% of hCD8^+^)	86.4 ± 3.3^b^	93.9 ± 1.6	ns	90.5 ± 1.8	ns
**Cytokines: ELISA**					
Plasma hIFNγ (ng/mL)	4.9 ± 1.0	4.2 ± 0.5	ns	3.6 ± 0.9	ns
Plasma hIL-6 (pg/mL)	8.9 ± 8.9	0.0 ± 0.0	ns	0.0 ± 0.0	ns

See also Figure 6.

a nonparametric (Kruskal-Wallis) test corrected by Dunn’s multiple comparisons test.

b *n* = 5 due to a technical issue during flow cytometry.

**Table:** Key resources table

REAGENT or RESOURCE	SOURCE	IDENTIFIER
**Antibodies**		
Human CD3 (OKT3)	Biolegend	317306; RRID:AB_571907
Human CD3 (OKT3)	Biolegend	317318; RRID:AB_1937212
Human CD3 (OKT3)	Biolegend	317336; RRID:AB_2561628
Human CD3 (UCH1)	Biolegend	300454; RRID:AB_2564149
Human CD3 (UCH1)	Biolegend	300458; RRID:AB_2564151
Human CD4 (OKT4)	Biolegend	317428; RRID:AB_1186122
Human CD8 (SK1)	Biolegend	344750; RRID:AB_2687201
Human CD11b (LM2)	Biolegend	393104; RRID:AB_2734451
Human CD19 (HIB19)	Biolegend	302212; RRID:AB_314242
Human CD25 (BC96)	Biolegend	302606; RRID:AB_314276
Human CD25 (BC96)	Biolegend	302622; RRID:AB_493755
Human CD45 (HI30)	Biolegend	304028; RRID:AB_893338
Human CD69 (FN50)	Biolegend	310910; RRID:AB_314845
Human CD301	Biolegend	354702; RRID:AB_11218998
Human EpCAM	Biolegend	324214; RRID:AB_2098808
Human HLA-A,B,C (W6/32)	Biolegend	311438; RRID:AB_2566306
Human PD-1 (EH12.2H7)	Biolegend	329906; RRID:AB_940483
Human TIM-3	Biolegend	345026; RRID:AB_2565717
Human TCR α/β	Biolegend	306706; RRID:AB_314644
Human IL-17	Biolegend	512702; RRID:AB_1027616
Human IL-10	Biolegend	506802; RRID:AB_315452
Human IFNγ	BD Biosciences	551221; RRID:AB_394099
Human IL-17, biotin	Biolegend	518902; RRID:AB_2561347
Human IL-10, biotin	Biolegend	501502; RRID: AB_315178
Human IFNγ, biotin	BD Biosciences	554550; RRID AB_395472
Human FOXP3 (236A/E7)	Thermo Fisher	17-4777-42; RRID:AB_10804651
Human CD3 (OKT3), Functional Grade	Thermo Fisher	16-0037-81; RRID:AB_468854
Human CD28 (CD28.2), Functional Grade	Thermo Fisher	16-0289-81; RRID:AB_468926
Human Cell-Surface Vimentin (CSV)	Abnova	H00007431-MA08
Mouse CD3 (17A2)	Biolegend	100236; RRID:AB_2561456
Mouse CD4 (RM4-5)	Biolegend	100540; RRID:AB_893326
Mouse CD4 (RM4-5)	Biolegend	100512; RRID:AB_312715
Mouse CD45 (30-F11)	Biolegend	103116; RRID:AB_312981
Mouse CD45 (30-F11)	Biolegend	103128; RRID:AB_493715
Mouse B220 (RA3-6B2)	Thermo Fisher	12-0452-82; RRID:AB_465671
Mouse CD8 (53-6.7)	Thermo Fisher	12-0081-81; RRID:AB_465529
Mouse CD19 (1D3)	Thermo Fisher	25-0193-82; RRID:AB_657663
Mouse IgM-HRP	Thermo Fisher	PA1-84383; RRID: AB_934032
Mouse IgG-HRP	Thermo Fisher	62-6520; RRID: AB_2533947
6x Histidine (AD1.1.10)	Abcam	ab117512; RRID: AB_10972195
6x Histidine-HRP	Abcam	ab1187; RRID: AB_298652
Anti-PHA-L	EY	AL-1801-2
**Biological samples**		
OVJ-1	Experimental Tissue Shared Resource, UCI	N/A
OVJ-2	Experimental Tissue Shared Resource, UCI	N/A
Patient derived metastatic Ewing’s Sarcoma	Experimental Tissue Shared Resource, UCI	31461
Patient derived metastatic Colon Adenocarcinoma	Experimental Tissue Shared Resource, UCI	31004
Colorectal Carcinoma Progression TMA	Cooperative Human Tissue Network, University of Virginia	CHTN_CRC2
Normal human tissue microarray	BioMAx	FDA999u
Normal human tissue microarray	BioMAx	FDA999w
Normal human tissue microarray	US Biolabs	FDA999-1
Prostate cancer microarray	Novus Biologicals	NBP2-30169
Breast cancer microarray	BioMAx	BR1005b
**Chemicals, peptides, and recombinant proteins**		
Tn antigen	Sigma	53886
GalNAc	Sigma	A2795
GlcNAc	Wellesley Therapeutics	Ultimate Glucosamine^®^
Kifunensine	GlycoSyn	FC-034
D-Luciferin	PerkinElmer	122799
GlyTR gene synthesis and plasmids	Genscript/ProBio	This paper
CD301-6xHistidine	R&D Systems	4888-CL-050
L-PHA, Fluorescein	Vector Laboratories	FL-1111; RRID: AB_2336655
L-PHA-Biotin	Vector Laboratories	B-1115; RRID: AB_2336654
VVA, Fluorescein	Vector Laboratories	FL-1231; RRID: AB_2336856
ConA, Fluorescein	Vector Laboratories	FL-1001-25; RRID: AB_2336348
Streptavidin-DyLight 649	Vector Laboratories	SA-5649; RRID: AB_2336421
Streptavidin-HRP	Biolegend	405210
Annexin V	Biolegend	640950; RRID: AB_2721650
Human IL-2	ThermoFisher	200-02-10UG
Human TGF-beta 1	ThermoFisher	100-21-10UG
Human IFNγ	Biolegend	570209
Human IL-10	Biolegend	571009
Human IL-17	Biolegend	570509
Human IL-6	Biolegend	570809
Cas-9 nuclease	Genscript	Z03469
Hematoxylin	Cell Signaling	14166
**Critical commercial assays**		
VivoTag 680XL Protein Labeling Kit	PerkinElmer	NEV11118
Alexa Fluor^™^ 647 Protein Labeling Kit	Thermo Fisher	A20173
FluoReporter^™^ FITC Protein Labeling Kit	Thermo Fisher	F6434
MycoAlert^™^ mycoplasma testing Kit	Lonza	LT07-218
MycoAlert^™^ Assay Control Set	Lonza	LT07-518
**Experimental models: Cell lines**		
Jurkat (Clone: E6-1)	ATCC	TIB-152
CD3-deficient Jurkat (Clone: J.RT3-T3.5)	ATCC	TIB-153
GnT KO HEK293S	ATCC	CRL-3022
Raji	ATCC	CCl-86
RPMI8226	ATCC	CRM-CCL-155
THP-1	ATCC	TIB-202
H1975	ATCC	CRL-5908
HeLa	ATCC	CCL-2
Hs766T	ATCC	HTB-134
HepG2	ATCC	HB-8065
Kasumi-1	ATCC	CRL-2724
RS4;11	ATCC	CRL-1873
MCF-7	ATCC	HTB-22
PL-21	DSMZ	ACC 536
JIMT-1	DSMZ	ACC 589
K562	David Fruman lab	N/A
MM1R	David Fruman lab	N/A
MM1S	David Fruman lab	N/A
K562	David Fruman lab	N/A
MM1R	David Fruman lab	N/A
MM1S	David Fruman lab	N/A
SU-DHL-6	David Fruman lab	N/A
MDA-MB-231F	Olga Razorenova lab	N/A
MDA-MB-231F-MI^−^ (Clone 2, 5, 11, 12)	Synthego and this paper	N/A
MDA-MB-231F-MI^−^ (Clone B2, MGAT5-)	Synthego	N/A
MDA-MB-231F-MI^−^C^−^ (Clone 4, COSMC KO)	This paper	N/A
Hey	Wenqi Wang lab	N/A
SKOV3	Wenqi Wang lab	N/A
SKOV3F-MI^−^	This paper	N/A
MeWo	Anand Ganesan lab	N/A
A375	Anand Ganesan lab	N/A
Panc1	Marian Waterman lab	N/A
DLD-1	Marian Waterman lab	N/A
LoVo	Marian Waterman lab	N/A
SW480	Marian Waterman lab	N/A
PC3	Mei Kong lab	N/A
A549	Janet Baulch	N/A
D54	Daniela Bota lab	N/A
U251	Daniela Bota lab	N/A
T98G	Daniela Bota lab	N/A
Patient derived Glioblastoma (DB93)	Daniela Bota lab	N/A
Patient derived Ovarian cancer stem cells	Celprogen Inc.	36113-40
Patient derived Breast cancer stem cells	Celprogen Inc.	36102-29
Patient derived lung cancer stem cells	Celprogen Inc.	36107-34
ExpiCHO-S	ThermoFisher	A29127
Mgat1-deficient ExpiCHO-S	This paper	N/A
Gale-deficient ExpiCHO-S	This paper	N/A
Human renal epithelial cells	ScienCell	4120
Human renal proximal tubular epithelial cells	ScienCell	4100
Human pulmonary alveolar epithelial cells	ScienCell	3200
Human hepatocytes	ScienCell	5200
Human brain vascular adventitial fibroblast	ScienCell	1110
Human prostate epithelial cells	ScienCell	4410
Human colonic epithelial cells	ScienCell	2950
**Experimental models: Organisms/strains**		
NOD.Cg-Prkdc^scid^ Il2rg^tm1Wjl^/SzJ	Jackson laboratory	005557
CD34+ Humanized NSG	Jackson laboratory	N/A
NOD.Cg-Prkdc^scid^ H2-K1^b-tm1Bpe^ H2-Ab1^g7-em1Mvw^H2-D1^b-tm1Bpe^ Il2rg^tm1Wjl^/SzJ	Jackson laboratory	025216
C57BL/6J	Jackson laboratory	000664
**Software and algorithms**		
Gensys Imaging analysis	Gensys	N/A
Flowjo	FlowJo LLC	N/A
GraphPad Prism	GraphPad	N/A
QuPath	QuPath	N/A
Xenogen IVIS Living Image 4.5.5	PerkinElmer	N/A
Aura Imaging software	Spectral Imaging	N/A
Microsoft Excel	Microsoft	N/A
Microsoft PowerPoint	Microsoft	N/A
Biorender	biorender.com	N/A

## Highlights

- “Velcro-like” lectin targeting of high-density carbohydrate antigens in diverse cancers
- High-avidity binding directs T cells to cancer but not low-density normal cells
- Checkpoint inhibition of β1,6-branching overcomes immunosuppressive tumor environments
- High-potency pan-cancer immunotherapy without “on-target, off-cancer” toxicity
