# Supplementary material for: Safe immunosuppression-resistant pan-cancer immunotherapeutics by velcro-like density-dependent targeting of tumor-associated carbohydrate antigens
Source: Cell. Author manuscript; Available in PMC 2026 Jan 5. (PMC12767472; doi:10.1016/j.cell.2025.09.001)
Supplement: Sup Tables 1 - 3 [file NIHMS2120256-supplement-Sup_Tables_1_-_3.pdf]

Cell, Volume *188*

Supplemental information

Safe immunosuppression-resistant pan-cancer immunotherapeutics by velcro-like density-dependent targeting of tumor-associated carbohydrate antigens

Raymond W. Zhou, Paresh Kumar Purohit, Jai Hyun Kim, Sung-Uk Lee, Nicole Burshteyn, Delia Tifrea, Andres Cordon, Ani Grigorian, Barbara L. Newton, Robert A. Edwards, and Michael Demetriou

Table S1. Clinical information on patient derived cancers, related to Figure S1 and Figure 5.

Cancer Type	Age	Sex	Previous Surgery	Previous Chemo/Radio	Metastases
Ovarian<br>(OVJ-1, resected 10/10/2022)	63	Female	Post chemo TAH, BSO, total omectomy & resection of peritoneal, sigmoid mesenteric mets	Pre resection Carbo/Taxol X 4 following discovery of PEUA and diagnostic laparoscopy with positive biopsies of nodules	Omentum, Peritoneum, Sigmoid mesentery, Left mesosalpinx, bilateral para ovarian tissue
Ovarian<br>(OVJ-2, resected 10/12/2022)	72	Female	Post-chemo BSO, pelvic peritonectomy, appendectomy, total omentectomy, multi peritoneal implant excisions/ablation & LN dissection.	Carbo/Taxol X 8	Pre chemo peritoneal carcinomatosis<br>Post chemo residual or recurrent bilateral ovarian carcinoma
Glioblastoma<br>IDH-wildtype (DB93)	58	Male	NA	NA	NA
Ewing Sarcoma<br>(31461 – lung met resected 1/27/2025)	30	Male	Left femur sarcoma resection with allograft and fibula reconstruction (10/29/2021).<br>Left lower lobe wedge resection for mets (1/22/2024)	Post initial resection<br>4 cycles VIT consolidated with SBRT following lung wedge resection (8/27-10/29, 2024).	One LLL lung met pre VIT and SBRT<br>Post one LLL lung met and two LUL lung mets
Colon carcinoma<br>(31004 – liver met resected 11/20/2024)	72	Female	Primary Resection (May 2022)<br>R lung lobectomy (June 2023)	Xelox (Dec 2022) & FOLFIRI+Bev (Jan 2024)	Lung, Liver
Human Ovarian Cancer Stem Cell<br>(Positive markers: CD44, CD133, SSEA 3/4, Oct4, Tumorigenicity (< 1000 cells), Alkaline Phosphatase, Aldehyde Dehydrogenase, Telomerase, Sox2, Nanog, cKit, Nestin, Lin28)	NA	NA	NA	NA	Stage 4
Human Breast Cancer Stem Cell<br>(Positive markers: CD133, CD44, SSEA3/4, Oct4, Tumorigenicity (<1000 cells), Alkaline Phosphatase, Aldehyde Dehydrogenase, Telomerase, Sox2)	NA	NA	NA	NA	Stage 4
Human Lung Cancer Stem Cell<br>(Positive markers: CD 133, CD43, SSEA3/4, Oct4, Tumorigenicity (<1000 cells), Alkaline Phosphatase, Aldehyde Dehydrogenase, Telomerase)	NA	NA	NA	NA	Stage 4

BSO - Bilateral salpingo-oophorectomy; TAH - total abdominal hysterectomy; Carbol – Carboplatin; Taxol – Placitaxel; VIT - Vincristine, Irinotecan and Temozolomide; SBRT - Stereotactic Body Radiation Therapy; LLL – left lower lobe, LUL – left upper lobe; Xelox - Capecitabine (Xeloda) and Oxaliplatin; FOLFIRI+Bev - Leucovorin (Folinic acid), Fluorouracil, and Irinotecan plus Bevacizumab; PEUA- Posterior Extrauterine Adhesion

Table S2. Toxicity assessment of GlyTR1 in CD34⁺ humanized NSG mice, related to Figure 6 and S7.

	CD34+ HuNSG mice						
	PBS (mean± SEM) n=3	GlyTR1, 2.5 µg (mean ± SEM) n=3	Statistical significance	GlyTR1, 5.0 µg (mean ± SEM) n=3	Statistical significance	GlyTR1, 10 µg (mean ± SEM) n=3	Statistical significance
**Chemistry**							
Total protein (g/dL)	5.2	5.2	NA	5.5	NA	5.3	NA
Albumin (g/dL)	2.9	2.8	NA	3.1	NA	3	NA
Globulin (g/dL)	2.3	2.4	NA	2.4	NA	2.3	NA
AST (IU/L)	136	93	NA	131	NA	115	NA
ALT (IU/L)	25	31	NA	31	NA	22	NA
ALK Phos (IU/L)	71	54	NA	53	NA	46	NA
T. Bilirubin (mg/dL)	0.2	0.2	NA	0.2	NA	0.2	NA
BUN (mg/dL)	19	23	NA	22	NA	21	NA
Creatinine (mg/dL)	0.2	0.2	NA	0.2	NA	0.2	NA
Sodium (mEq/dL)	148	151	NA	151	NA	149	NA
Chloride (mEq/dL)	113	113	NA	113	NA	114	NA
Potassium (mEq/dL)	5.6	5.4	NA	5.3	NA	5.4	NA
Calcium (mg/dL)	9.5	9.8	NA	10	NA	10	NA
Phosphorus (mg/dL)	8.4	8.5	NA	8.3	NA	7.8	NA
Glucose (mg/dL)	166	200	NA	203	NA	185	NA
Amylase (IU/L)	598	609	NA	646	NA	594	NA
Precision PSL (IU/L)	20	21	NA	22	NA	21	NA
TSH (ng/mL)	0.04	0.04	NA	0.04	NA	0.06	NA
Creatine phospho-kinase (CPK) (IU/L)	612	325	NA	584	NA	535	NA
Cholesterol (mg/dL)	71	81	NA	80	NA	64	NA
**Complete Blood Count**							
RBC (x 10^6/uL)	4.2 ± 0.7	4.7 ± 0.7	ns	4.4 ± 0.3	ns	3.7 ± 0.7	ns
Hemoglobin (g/dL)	9.4 ± 1.1	10.6 ± 1.3	ns	10.0 ± 0.6	ns	8.4 ± 1.3	ns
Hematocrit (%)	27.6 ± 3.8	32.2 ± 4.0	ns	30.0 ± 1.4	ns	25.2 ± 3.7	ns
WBC (x 10^3/uL)	0.9 ± 0.2	1.4 ± 0.3	ns	1.9 ± 0.8	ns	1.6 ± 0.4	ns
Platelets (x 10^3/uL)	1049 ± 129	1166 ± 8.5	ns	1198 ± 80.2	ns	1028 ± 110	ns
Lymphocytes (%)	57.1#	57.5 ± 14.7	NA	53.0 ± 2.5	NA	52.8 ± 7.8	NA
Monocytes (%)	8.5#	9.3 ± 1.1	NA	9.6 ± 0.5	NA	8.5 ± 2.6	NA
Granulocytes (%)	34.4#	33.3 ± 13.6	NA	37.6 ± 1.9	NA	38.7 ± 5.6	NA
**Spleen: Flow Cytometry**							
hCD45+ splenocytes (x 10^6)	9.0 ± 2.6	14.5 ± 4.1	ns	20.0 ± 12.9	ns	10.0 ± 1.2	ns
$hCD4^+$ (% of $hCD45^+$)	21.0 ± 1.6	20.3 ± 9.9	ns	19.1 ± 7.9	ns	30.2 ± 14.5	ns
$hCD8^+$ (% of $hCD45^+$)	11.9 ± 2.0	8.6 ± 3.3	ns	9.0 ± 4.3	ns	17.3 ± 8.7	ns
$hCD19^+$ (% of $hCD45^+$)	54.5 ± 7.6	61.3 ± 13.1	ns	61.0 ± 13.3	ns	41.7 ± 24.1	ns
$Foxp3^+$ (% of $hCD4^+$)	7.9 ± 0.8	6.1 ± 1.7	ns	8.3 ± 5.3	ns	8.2 ± 4.0	ns
$hCD25^+$ (% of $hCD4^+$)	1.8 ± 0.3	2.7 ± 0.5	ns	2.9 ± 1.3	ns	3.5 ± 0.6	ns
$hCD69^+$ (% of $hCD4^+$)	29.3 ± 4.7	28.0 ± 5.3	ns	29.5 ± 7.2	ns	18.5 ± 6.6	ns
$hCD69^+$ (% of $hCD8^+$)	22.2 ± 7.5	30.4 ± 4.5	ns	35.6 ± 4.9	ns	26.4 ± 7.1	ns
$hCD25^+$ (% of $hCD8^+$)	1.1 ± 0.3	2.6 ± 1.1	ns	2.7 ± 0.5	ns	3.1 ± 0.4	ns
$hPD1^+$ (% of $hCD4^+$)	46.5 ± 10.9	48.7 ± 13.2	ns	46.8 ± 7.4	ns	57.0 ± 8.2	ns
$hPD1^+$ (% of $hCD8^+$)	42.4 ± 24.6	61.3 ± 3.7	ns	61.3 ± 5.1	ns	65.0 ± 7.8	ns
**Cytokines: ELISA**							
Plasma hIFNγ (ng/mL)	0.0 ± 0.0	93.6 ± 93.6	ns	0.0 ± 0.0	ns	0.0 ± 0.0	ns
Plasma hIL-6 (pg/mL)	0.0 ± 0.0	13.6 ± 13.6	ns	0.0 ± 0.0	ns	0.0 ± 0.0	ns

#: only one meaningful data point was collected/shown by CBC analyzer.

Table S3. PHA lectins did not induce acute hyper-sensitivity in human, related to Figure 6.

Patients treated	PHA type/source	IV Dose	Adverse Effects	Clinical Benefit	Reference
4	PHA-P (Wellcome)	50mg qd x 7	Minor allergic phenomena<br>Pain in thorax/leg bones	Yes	Humble Nature 1963
2	PHA-P (Wellcome)	50mg qd x 7	None reported	Yes	Humble Lancet 1964
1	PHA-P (Wellcome)	50mg qd x 6	None reported	No	Fleming Lancet 1964
6	PHA-P (Wellcome)	50mg qd x 7	No severe systemic reactions<br>Superficial phlebitis in 2 patients, one of which also c/o body ache with slight fever.	No	Retief Lancet 1964
1	PHA-P (Wellcome)	50mg qd x 7 then<br>50mg qd x 3	None reported	Yes	Baker Lancet 1965
3	PHA-P (Wellcome)	1) 50mg qd x 7<br>2) 2 courses of 50mg qd x 7<br>3) 3 courses of 50mg qd x 7	None reported	2 of 3	Gruenwald Lancet 1965
1	PHA-P (Wellcome)	50mg qd x 7 then<br>50mg qd x 3 then<br>50mg qd x 7	Mild side effects of diffuse sensation of heat after each injection and mild fever (99F) after first injection	No	Mehra Lancet 1965
1	PHA-P (Wellcome)	50mg qd x 7	Rigors after first two injections subsequently prevented by pre-medication with chlorpromazine.<br>Muscle and bone pain after 2nd injection.	No	Gurling Lancet 1965
12	PHA-P (Wellcome)	50mg qd x 7	“In no case was any harmful effect noted”	4 of 12	Allison Scott Med J 1965
1	PHA-P (Wellcome)	250mg over 50hrs	‘No particular side effects’	Yes	Buschor Lancet 1966
3	PHA-P (Wellcome)	1) 50mg qd x 11<br>2) 50mg qd x 21 in four series<br>3) 50mg qd x 18 in three series	None reported	1-2 of 3	Askoy Lancet 1966
3	PHA-P (Wellcome)	1) 50mg qd x 7<br>2) 2 courses of 50mg qd x 5<br>3) 50mg qd x 14	None reported	No	Hayes Blood 1966
6	PHA-P (Wellcome)	1-5) 50mg qd x 7<br>6) 2 courses of 50mg qd x 7	None reported	2 of 6	Catovsky Lancet 1967
3	PHA-M (Difco)	1) 100mg qd x 1<br>2-3) 50mg q2-3d x 7	None reported		Astaldi Int Arch Allergy 1967
